# Expression Profiles of Fatty Acid Transporters and the Role of n-3 and n-6 Polyunsaturated Fatty Acids in the Porcine Endometrium

**DOI:** 10.3390/ijms252011102

**Published:** 2024-10-16

**Authors:** Agnieszka Blitek, Magdalena Szymanska

**Affiliations:** Institute of Animal Reproduction and Food Research, Polish Academy of Sciences, 10-748 Olsztyn, Poland; m.szymanska@pan.olsztyn.pl

**Keywords:** pig, endometrium, estrous cycle, pregnancy, fatty acid transporters, polyunsaturated fatty acids, prostaglandin synthesis

## Abstract

Fatty acids (FAs) are important for cell membrane composition, eicosanoid synthesis, and metabolic processes. Membrane proteins that facilitate FA transport into cells include FA translocase (also known as CD36) and FA transporter proteins (encoded by *SLC27A* genes). The present study aimed to examine expression profiles of FA transporters in the endometrium of cyclic and early pregnant gilts on days 3 to 20 after estrus and the possible regulation by conceptus signals and polyunsaturated FAs (PUFAs). The effect of PUFAs on prostaglandin (PG) synthesis and transcript abundance of genes related to FA action and metabolism, angiogenesis, and immune response was also determined. Day after estrus and reproductive status of animals affected FA transporter expression, with greater levels of CD36, SLC27A1, and SLC27A4 observed in pregnant than in cyclic gilts. Conceptus-conditioned medium and/or estradiol-17β stimulated SLC27A1 and CD36 expression. Among PUFAs, linoleic acid decreased *SLC27A1* and *SLC27A6* mRNA expression, while arachidonic, docosahexaenoic, and eicosapentaenoic acids increased *SLC27A4* transcript abundance. Moreover, arachidonic acid stimulated *ACOX1*, *CPT1A*, and *IL1B* expression and increased PGE2 and PGI2 secretion. In turn, α-linolenic acid up-regulated *VEGFA, FGF2, FABP4,* and *PPARG* mRNA expression. These results indicate the presence of an active transport of FAs in the porcine endometrium and the role of PUFAs as modulators of the uterine activity during conceptus implantation.

## 1. Introduction

Nutrition has a major impact on many aspects of reproduction, and an imbalanced diet may negatively affect reproductive outcomes [1,2,3,4]. Among nutrients, lipids are of critical importance for cell differentiation and function, being the main energy source. A group of lipids known as polyunsaturated fatty acids (PUFAs) are essential constituents of cell membrane phospholipids that maintain cellular and organelle integrity [5]. PUFAs are precursors for eicosanoids and may affect enzymatic and transcriptional networks, leading to the regulation of gene expression, activation of intracellular signaling pathways, and modulation of angiogenesis and inflammatory responses [6,7,8,9,10,11].

PUFAs are classified into three groups: omega-3 (n-3), omega-6 (n-6), and omega-9 (n-9) based on the position of the first double bond relative to the methyl end of the molecule. Mammals are not able to synthesize de novo n-6 and n-3 PUFAs as they lack specific FA desaturase enzymes; thus, these FAs must be obtained from food. PUFAs from the n-6 series consist primarily of the precursor named linoleic acid (LA; C18:2n-6) and its long-chain derivative, arachidonic acid (ARA; C20:4n-6). PUFAs from the n-3 series include α-linolenic acid (ALA; C18:3n-3) and its long-chain derivatives, eicosapentaenoic acid (EPA; C20:5n-3) and docosahexaenoic acid (DHA; C22:6n-3). Dietary LA and ALA must be converted in the body to their long-chain metabolites to exert a full range of biological actions [12,13].

The transport of FAs into cells may occur via simple diffusion (mainly short- and medium-chain FAs) or by facilitated transport (long-chain FAs) [14,15]. Membrane proteins that participate in the transport of FAs include FA translocase (FAT; also known as CD36) and FA transport proteins (FATPs; encoded by *SLC27A* genes), which comprise six identified members, FATP1 to FATP6. Except for FATP5, whose expression in the body is restricted to the liver, CD36 and other FATPs are expressed with different intensities in various tissues, including the liver, kidney, muscle, adipose tissue, heart, and skin. Their expression has also been reported in the testis, uterus, and placenta [16].

The majority of data describing the active transport of FAs and its importance for cell functions is related to the well-described critical role of n-3 and n-6 PUFAs for embryo/fetus growth as well as in placenta formation and function [6,13,17,18]. CD36 and FATPs were localized on both the microvillous and basal membranes of the human placenta [19]. Moreover, the expression of FA transporters in the human placenta may be regulated by FAs [20], and FAs affect the viability, gene expression, and secretory activity of trophoblast cells [13,18,21,22]. Among livestock species, the regulation of FA transporter expression in the placenta has been reported for cattle [23], sheep [24], and pigs [25,26,27].

Much less data is available regarding the transport and function of FAs in the maternal endometrium. The uterine endometrium is a dynamic tissue that undergoes structural and functional changes during each estrous cycle to prepare for conceptus implantation. These changes include growth, differentiation, and regression periods, each associated with the modulation of gene expression, protein synthesis, and the activation of several intracellular pathways [28,29,30]. In the pig, most of these changes are regulated by ovarian steroids, mainly progesterone [31]. During pregnancy, however, developing conceptuses may modulate the endometrial milieu by secreting various biological molecules like prostaglandin E2 (PGE2), 17β-estradiol (E2), interferons, and cytokines to facilitate implantation [32,33,34]. Of interest, genes related to lipid biosynthesis, transport, and metabolism were among the differentially expressed genes in the endometrium of days 12 and 14 pregnant gilts compared with non-pregnant gilts [35,36]. Moreover, the endometrial *SLC27A1* gene, which encodes the FATP1 protein, has been selected as a candidate gene positively affecting pig litter size [37]. These results point to the possible important role of FA transport in endometrial preparation for conceptus implantation in the pig. We hypothesize that FA transporter expression in the endometrium differs in cyclic and pregnant gilts and that FAs may affect endometrial gene expression for successful embryo-maternal communication and pregnancy establishment. Thus, the present study was conducted (1) to examine the mRNA and protein expression of CD36 and SLC27A in the endometrium of cyclic and early pregnant gilts and (2) to analyze the effect of conceptus products on the expression of selected FA transporters in this tissue. Moreover, (3) we determined whether PUFAs of the n-6 and n-3 series may influence FA transporter expression, PGE2 and PGI2 formation, and the mRNA expression of selected genes in the endometrial tissue of early pregnant gilts. Genes encoding factors involved in intracellular FA binding (*FABP3*, *FABP4*, *FABP5*), action (*PPARA*, *PPARD*, *PPARG*), and metabolism (*ACOX1*, *CPT1A*) as well as immune response (*IL1B*, *IL6*, *TNF*) and angiogenesis (*VEGFA*, *FGF2*) were chosen for mRNA analysis (full names of genes are specified in Supplementary Table S1).

## 2. Results

### 2.1. The mRNA Expression of FA Transporters in the Endometrium of Cyclic and Early Pregnant Gilts

**Figure 1:**
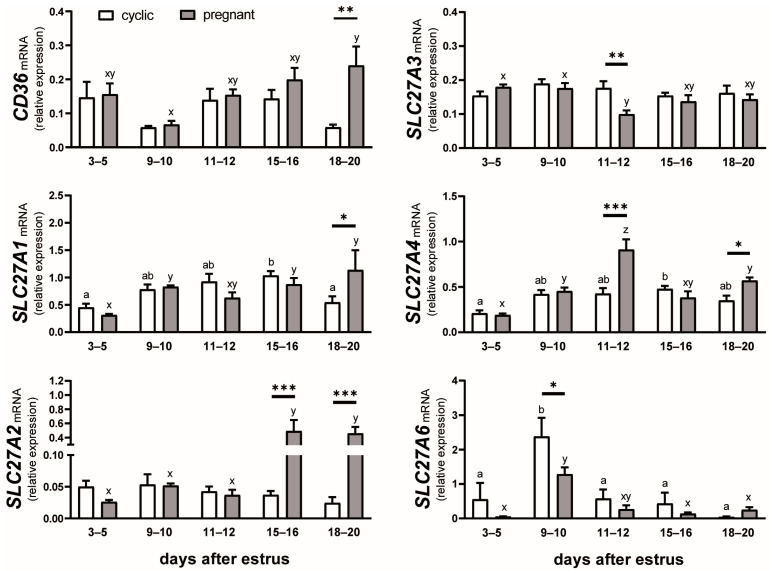
Expression of *CD36*, *SLC27A1*, *SLC27A2*, *SLC27A3*, *SLC27A4*, and *SLC27A6* mRNA in the endometrium of cyclic (white bars) and pregnant (grey bars) gilts. Values from real-time PCR were normalized to geometric averaging of glyceraldehyde-3-phosphate dehydrogenase (*GAPDH*) and hypoxanthine phosphoribosyltransferase 1 (*HPRT1*) mRNA expression. Data are expressed as means ± SEM (*n* = 5–6). Bars marked with various letters differ among groups (a, b—cyclic; x, y, z—pregnant). Asterisks specify differences between cyclic and pregnant animals on particular days after estrus (*, *p* < 0.05; **, *p* < 0.01; ***, *p* < 0.001).

The relative abundance of *CD36* transcripts was affected by the day after estrus (*p* = 0.03) and the reproductive status of animals (*p* = 0.02; Figure 1). *CD36* mRNA expression was greater on days 18–20 of pregnancy compared with days 9–10 (*p* < 0.01; 3.6-fold increase) and also compared with the respective days of the estrous cycle (*p* < 0.01; 4-fold increase).

The day after estrus (*p* < 0.001) and the day by estrus interaction (*p* = 0.01) affected the expression of *SLC27A1* in the endometrium (Figure 1). The transcript abundance of *SLC27A1* increased more than doubled between days 3–5 and 15–16 in both cyclic and pregnant gilts (*p* < 0.05), followed by a decrease on days 18–20 in cyclic (*p* < 0.05) but not pregnant animals. On days 18–20, *SLC27A1* mRNA expression was 2-fold greater in pregnant than in cyclic gilts (*p* < 0.05).

The abundance of *SLC27A2*, *SLC27A3*, and *SLC27A4* transcripts was affected by the day after estrus (*p* = 0.0001, *p* = 0.04, and *p* < 0.0001, respectively), reproductive status of animals (*p* < 0.0001, *p* = 0.04, and *p* = 0.0012, respectively), and the day by reproductive status interaction (*p* < 0.0001, *p* = 0.02, and *p* < 0.0001, respectively; Figure 1). The mRNA expression of *SLC27A2* was more than 10-fold greater on days 15–16 and 18–20 of pregnancy compared with days 3–12 (*p* < 0.001) and also compared with the respective days of the estrous cycle (*p* < 0.001). By contrast, the mRNA expression of *SLC27A3* decreased on days 11–12 of pregnancy (*p* < 0.01 compared with days 3–10) and was lower in pregnant than cyclic animals (*p* < 0.01). *SLC27A4* showed a dynamic profile of expression in pregnant animals with a progressive increase between days 3–5 and 11–12 (*p* < 0.001), followed by a decrease on days 15–16 (*p* < 0.001; 60% reduction compared with days 11–12) with no changes thereafter. Greater expression of *SLC27A4* mRNA in pregnant than in cyclic gilts was detected on days 11–12 (*p* < 0.001; 2-fold increase) and 18–20 (*p* < 0.05; 1.6-fold increase) after estrus.

The relative abundance of *SLC27A6* transcripts was affected by the day after estrus (*p* < 0.001) and the reproductive status of animals (*p* = 0.02; Figure 1). *SLC27A6* mRNA expression was greater on days 9–10 compared with days 3–5, 15–16, and 18–20 in both cyclic (*p* < 0.001) and pregnant (*p* < 0.05) animals. The effect of reproductive status was detected on days 9–10 after estrus when cyclic gilts showed almost 2-fold greater abundance of *SLC27A6* transcripts than pregnant animals (*p* < 0.05).

### 2.2. The Protein Expression of FA Transporters in the Endometrium of Cyclic and Early Pregnant Gilts

**Figure 2:**
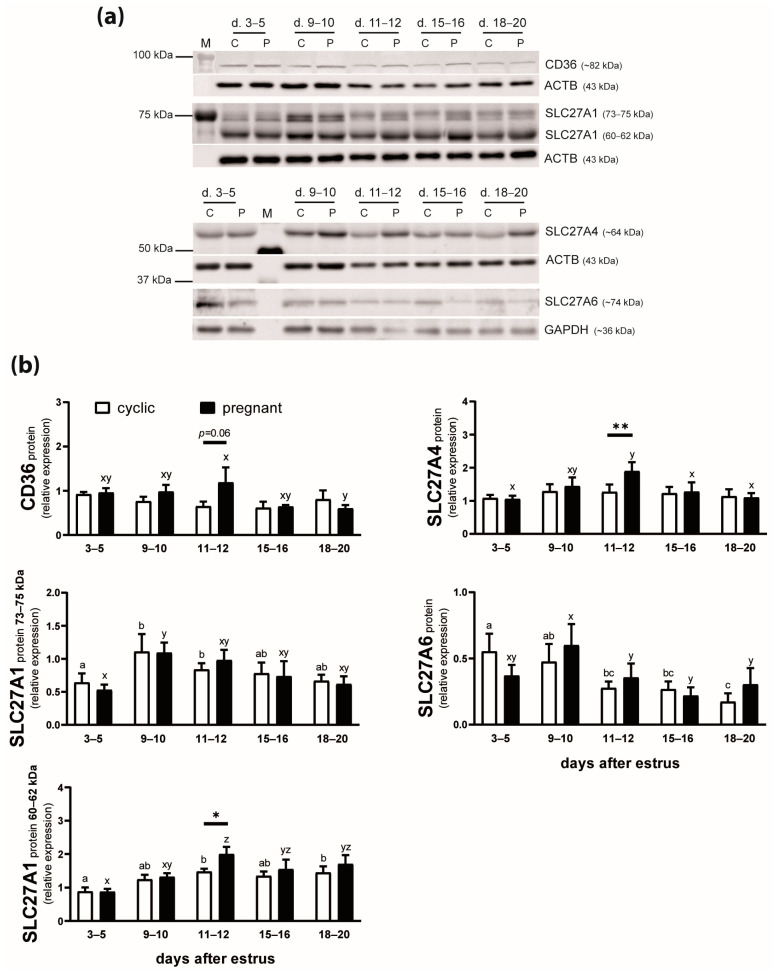
Expression of CD36, SLC27A1, SLC27A4, and SLC27A6 proteins in the endometrium of cyclic (white bars) and pregnant (black bars) gilts. Representative blots are presented (panel **a**: C—cyclic, P—pregnant, M—marker, d.—day after estrus; full blots are included in Supplementary Figures S1 and S2). Values from densitometric analyses of bands were normalized to β-actin (ACTB) or glyceraldehyde-3-phosphate dehydrogenase (GAPDH) and expressed as means ± SEM (*n* = 5; panel **b**). Bars marked with various letters differ among groups (a, b, c—cyclic; x, y, z—pregnant). Asterisks specify the differences between cyclic and pregnant animals on particular days after estrus (*, *p* < 0.05; **, *p* < 0.01).

The expression of CD36 protein was affected by the reproductive status of animals (*p* = 0.002) but not the day after estrus (*p* = 0.16) or the day by reproductive status interaction (*p* = 0.12; Figure 2). In pregnant gilts, CD36 expression decreased by 50% between days 11–12 and 18–20 (*p* ≤ 0.05). Moreover, the protein level of this FA transporter tended to be greater in pregnant than in cyclic animals on days 11–12 after estrus (*p* = 0.06).

SLC27A1 protein in the endometrium was detected as two bands of 73–75 and 60–62 kDa (Figure 2a). The expression of both isoforms of SLC27A1 protein was affected by the day after estrus (*p* = 0.01; Figure 2b). The expression of the 73–75 kDa SLC27A1 increased in endometrial tissue on days 9–10 compared with days 3–5 (*p* < 0.05) in both cyclic and pregnant gilts. The 60–62 kDa SLC27A1 expression was greater on days 11–12 than on days 3–5 of the estrous cycle (*p* < 0.05) and pregnancy (*p* < 0.01). Moreover, the protein level of this isoform was elevated in pregnant compared with cyclic animals on days 11–12 after estrus (*p* < 0.05; 1.4-fold increase).

The expression of SLC27A4 protein was affected by the day after estrus (*p* = 0.03) and the day by reproductive status interaction (*p* = 0.02; Figure 2). Greater expression of this protein was observed on days 11–12 of pregnancy compared with days 3–5, 15–16, and 18–20 (*p* < 0.01) and also compared with respective days of the estrous cycle (*p* < 0.01).

SLC27A6 protein expression in endometrial tissue was affected by the day after estrus (*p* = 0.02; Figure 2). In cyclic gilts, SLC27A6 protein content was lower on days 11 to 20 compared with days 3–5 (*p* < 0.05). A similar profile of SLC27A6 expression was observed in pregnant gilts, with almost 2-fold lower levels detected on days 11 to 20 compared with days 9–10 (*p* < 0.01).

The application of various anti-SLC27A2 and anti-SLC27A3 antibodies did not result in the detection of specific bands. Therefore, CD36, SLC27A1, SLC27A4, and SLC27A6 proteins (presented in Figure 2) were examined during further in vitro experiments.

### 2.3. Localization of FA Transporters in the Endometrium

**Figure 3:**
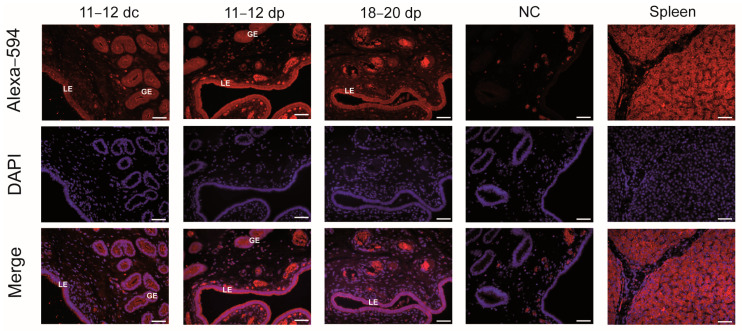
CD36 protein localization in the endometrium of days 11–12 cyclic and days 11–12 and 18–20 pregnant gilts using immunofluorescence technique. Tissue sections were incubated with Alexa–594-labeled secondary antibody (red) and counterstained with diamidino-2-phenylindole (DAPI; blue) to visualize nuclei. For negative control (NC), primary antibodies were replaced with rabbit IgG. Spleen was used as a positive control tissue for CD36 protein expression. dc: days of the estrous cycle; dp: days of pregnancy; LE: luminal epithelium; GE: glandular epithelium. Scale bars, 50 μm.

The use of the immunofluorescence (IF) procedure allowed for the localization of CD36 protein in the luminal and glandular epithelium of the porcine endometrium as well as in the spleen, used as a positive tissue for CD36 presence (Figure 3).

**Figure 4:**
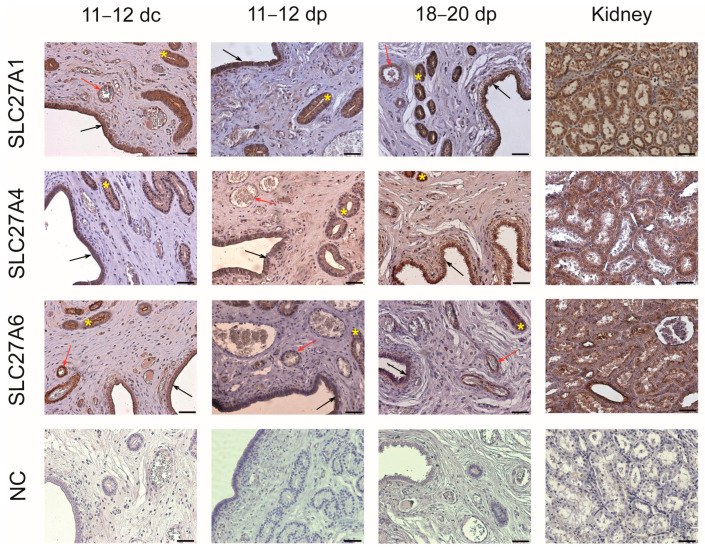
SLC27A1, SLC27A4, and SLC27A6 protein localization in the endometrium of days 11–12 cyclic and days 11–12 and 18–20 pregnant gilts using immunohistochemical analysis. Tissue sections were counterstained with Mayer’s hematoxylin (blue staining) to visualize nuclei. For negative control (NC), primary antibodies were replaced with rabbit IgG. Kidney tissue was used as a positive control for SLC27A protein expression. Black arrows—luminal epithelium, yellow asterisks—glandular epithelium, red arrows—blood vessels. dc: days of the estrous cycle; dp: days of pregnancy. Scale bars, 50 μm.

SLC27A1, SLC27A4, and SLC27A6 proteins were detected using immunohistochemical (IHC) analysis in the luminal and glandular epithelium of the endometrial tissue (Figure 4). Moreover, blood vessels showed strong positive staining for SLC27A1 and SLC27A6 presence. The kidney, used as a positive tissue for SLC27A protein expression, was visibly stained for all examined proteins.

### 2.4. Experiment 1. Effect of Conceptus Signals on the Expression of FA Transporters in the Endometrium

**Figure 5:**
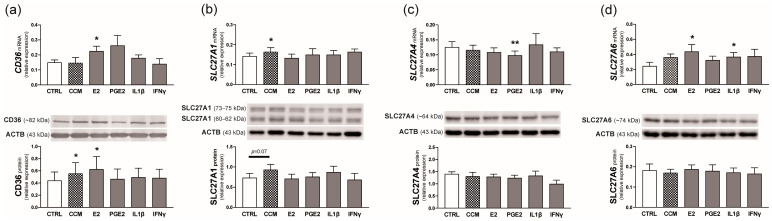
Effect of conceptus-conditioned medium (CCM), estradiol-17β (E2), prostaglandin E2 (PGE2), interleukin 1β (IL1β), and interferon γ (IFNγ) on the relative mRNA and protein expression of CD36 (**a**), SLC27A1 (**b**), SLC27A4 (**c**), and SLC27A6 (**d**) in the porcine endometrium. Values from real-time PCR were normalized to geometric averaging of glyceraldehyde-3-phosphate dehydrogenase (*GAPDH*) and hypoxanthine phosphoribosyltransferase 1 (*HPRT1*) mRNA expression. Values from densitometric analyses of bands were normalized to β-actin (ACTB). SLC27A1 protein was calculated as a sum of 73–75 and 60–62 kDa bands. Representative blots are presented (full blots are included in Supplementary Figures S3 and S4). All numerical data are expressed as means ± SEM (*n* = 6). Asterisks specify the differences compared with the control value (CTRL; *, *p* ≤ 0.05; **, *p* < 0.01).

The mRNA expression of *CD36* was stimulated by E2 (*p* < 0.05 compared with the control value; 1.5-fold increase), while the protein expression increased in the presence of conceptus-conditioned medium (CCM) or E2 in the incubation medium (*p* ≤ 0.05; Figure 5a).

The addition of CCM resulted in an elevated concentration of *SLC27A1* transcripts in the endometrial tissue (*p* < 0.05 compared with the control value) and tended to increase SLC27A1 protein level (*p* = 0.07; Figure 5b).

A lower concentration of endometrial *SLC27A4* transcripts was observed in the presence of PGE2 (*p* < 0.01 compared with the control value). SLC27A4 protein level, however, was unaffected by the examined factors (Figure 5c).

E2 and interleukin 1β (IL1β) stimulated *SLC27A6* mRNA expression in endometrial explants (*p* < 0.05; 1.8- and 1.5-fold increase, respectively). SLC27A6 protein, however, did not change in response to the applied treatments (Figure 5d).

### 2.5. Experiment 2. Effect of n-6 and n-3 PUFAs on FA Transporter Expression in the Endometrium

**Figure 6:**
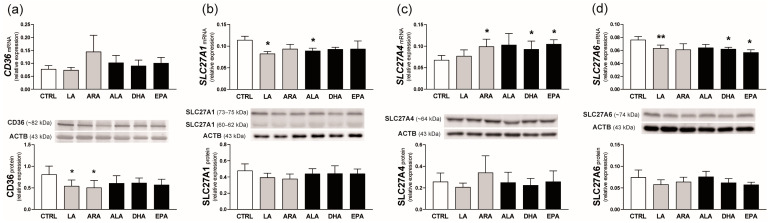
Effect of n-6 (linoleic acid [LA] and arachidonic acid [ARA]; grey bars) and n-3 (α-linolenic acid [ALA], docosahexaenoic acid [DHA], and eicosapentaenoic acid [EPA]; black bars) PUFAs on the relative mRNA and protein expression of CD36 (**a**), SLC27A1 (**b**), SLC27A4 (**c**), and SLC27A6 (**d**) in the endometrium. Values from real-time PCR were normalized to geometric averaging of glyceraldehyde-3-phosphate dehydrogenase (*GAPDH*) and hypoxanthine phosphoribosyltransferase 1 (*HPRT1*) mRNA expression. Values from densitometric analyses of bands were normalized to β-actin (ACTB). SLC27A1 protein was calculated as a sum of 73–75 and 60–62 kDa bands. Representative blots are presented (full blots are included in Supplementary Figures S5 and S6). All numerical data are expressed as means ± SEM (*n* = 5). Asterisks specify the differences compared with the control value (CTRL; *, *p* ≤ 0.05; **, *p* < 0.01).

Incubation of endometrial strips with PUFAs of the n-6 or n-3 series did not affect *CD36* mRNA expression; however, LA and ARA decreased CD36 protein content by about 35% (*p* < 0.05 compared with the control value; Figure 6a).

The presence of LA and ALA, but not their long-chain derivatives, in the incubation medium, reduced *SLC27A1* transcript concentration in the endometrial tissue (*p* < 0.05). Their effect on SLC27A1 protein level was not observed (Figure 6b).

The abundance of *SLC27A4* transcripts was stimulated by ARA, DHA, and EPA (*p* ≤ 0.05; 1.5-fold increase). SLC27A4 protein, however, did not change in the presence of PUFAs (Figure 6c).

*SLC27A6* mRNA expression was reduced by LA (*p* < 0.01) as well as DHA and EPA (*p* < 0.05) treatment of endometrial slices. Similar to SLC27A1 and SLC27A4, no changes in SLC27A6 protein content were detected (Figure 6d).

### 2.6. Experiment 3. Effect of n-6 and n-3 PUFAs on PGE2 and PGI2 Synthesis and the Concentration of Selected Transcripts in the Endometrium

**Figure 7:**
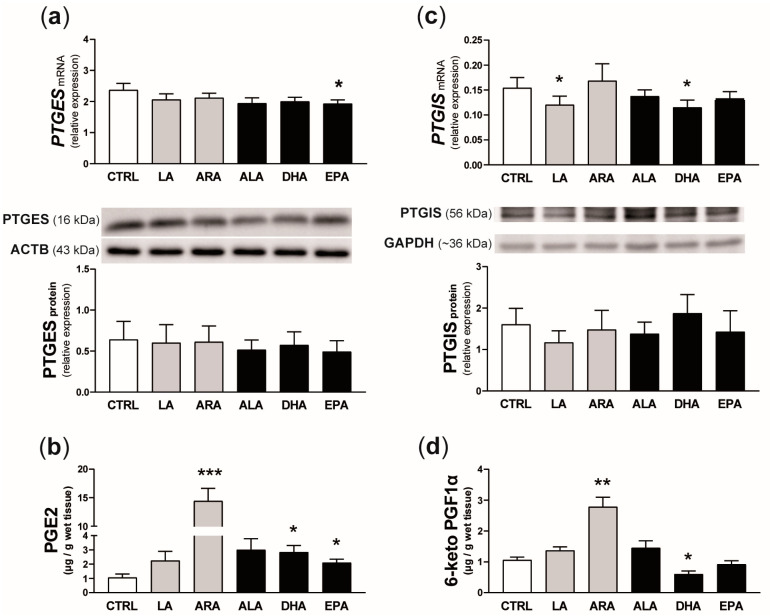
Effect of n-6 (linoleic acid [LA] and arachidonic acid [ARA]; grey bars) and n-3 (α-linolenic acid [ALA], docosahexaenoic acid [DHA], and eicosapentaenoic acid [EPA]; black bars) PUFAs on the relative mRNA and protein expression of prostaglandin E synthase (PTGES; **a**) and prostaglandin I2 synthase (PTGIS; **c**) in the endometrium as well as prostaglandin E2 (PGE2; **b**) and 6-keto prostaglandin F1α (6-keto PGF1α, a stable metabolite of PGI2; **d**) concentrations in the incubation medium. Values from real-time PCR were normalized to geometric averaging of glyceraldehyde-3-phosphate dehydrogenase (*GAPDH*) and hypoxanthine phosphoribosyltransferase 1 (*HPRT1*) mRNA expression. Values from densitometric analyses of bands were normalized to β-actin (ACTB) or GAPDH. Representative blots are presented (full blots are included in Supplementary Figure S7). All numerical data are expressed as means ± SEM (*n* = 5). Asterisks specify the differences compared with the control value (CTRL; *, *p* ≤ 0.05; **, *p* < 0.01; ***, *p* < 0.001).

*PTGES* mRNA expression in endometrial slices was reduced by the addition of EPA to the incubation medium (*p* = 0.05; Figure 7a), while LA and DHA decreased *PTGIS* transcript abundance (*p* < 0.05; Figure 7c). None of the examined PUFAs affected PTGES or PTGIS protein expression. In turn, concentrations of PGE2 and 6-keto PGF1α in the incubation medium increased 14 times (*p* < 0.001) and 2.6 times (*p* < 0.01), respectively, in the presence of ARA. Moreover, DHA and EPA stimulated PGE2 release into the medium (*p* < 0.05; more than 2-fold increase). By contrast, 43% lower concentrations of 6-keto PGF1α in the incubation medium were observed after the treatment of endometrial explants with DHA (*p* = 0.05).

**Figure 8:**
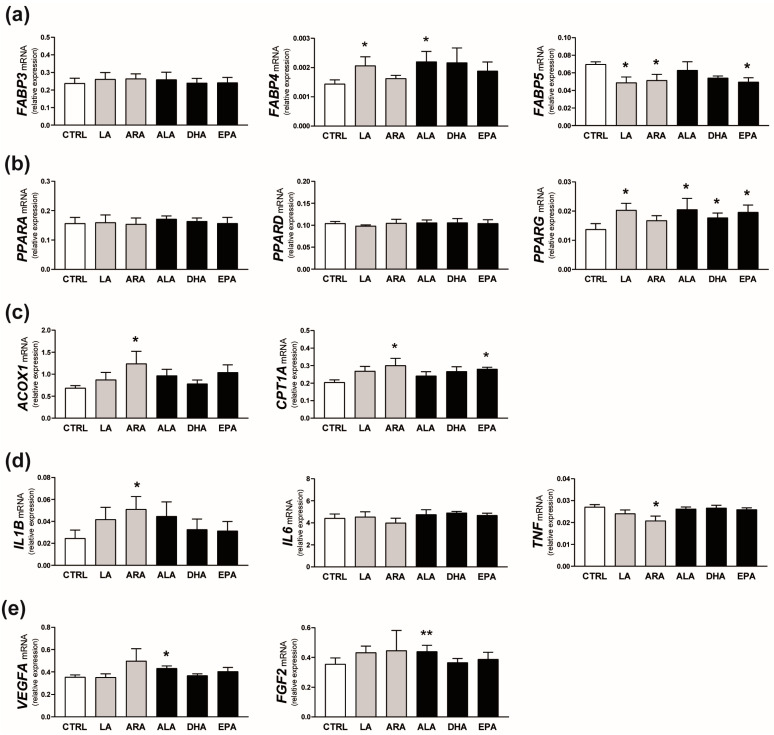
Effect of n-6 (linoleic acid [LA] and arachidonic acid [ARA]; grey bars) and n-3 (α-linolenic acid [ALA], docosahexaenoic acid [DHA], and eicosapentaenoic acid [EPA]; black bars) PUFAs on the relative mRNA expression of genes involved in fatty acid binding (*FABP3*, *FABP4*, *FABP5*; **a**), action (*PPARA*, *PPARD*, *PPARG*; **b**), and metabolism (*ACOX1*, *CPT1A*; **c**) as well as genes related to immune response (*IL1B*, *IL6*, *TNF*; **d**) and angiogenesis (*VEGFA*, *FGF2*; **e**) in the endometrium. Values from real-time PCR were normalized to geometric averaging of glyceraldehyde-3-phosphate dehydrogenase (*GAPDH*) and hypoxanthine phosphoribosyltransferase 1 (*HPRT1*) mRNA expression. Data are expressed as means ± SEM (*n* = 5). Asterisks specify the differences compared with the control value (CTRL; *, *p* ≤ 0.05; **, *p* < 0.01).

Examined PUFAs differentially affected the mRNA expression of genes related to FA binding, action, and metabolism in the endometrial tissue (Figure 8). The addition of LA and ALA to the incubation medium stimulated the mRNA expression of *FABP4* in endometrial explants compared with the control value (*p* ≤ 0.05; 1.5-fold increase). By contrast, *FABP5* mRNA expression was decreased by about 30% in response to LA, ARA, or EPA (*p* ≤ 0.05; Figure 8a). The abundance of *PPARG* but not *PPARA* or *PPARD* transcripts increased after the treatment of explants with LA, ALA, DHA, or EPA (*p* < 0.05; Figure 8b). Moreover, ARA stimulated *ACOX1* and *CPT1A* mRNA expression (*p* < 0.05; 1.8- and 1.5-fold increase, respectively) while EPA increased *CPT1A* mRNA expression in endometrial tissue (*p* < 0.05; 1.4-fold change; Figure 8c).

Among immune response-related transcripts examined, *IL6* mRNA abundance was not affected by PUFA presence in the incubation medium. The addition of ARA resulted in 2 times greater expression of *IL1B* and decreased expression of *TNF* compared with respective control values (*p* < 0.05; Figure 8d).

Exposure of endometrial explants to ALA increased concentrations of *VEGFA* and *FGF2* transcripts by about 20% (*p* < 0.05 and *p* < 0.01, respectively; Figure 8e).

## 3. Discussion

To the best of our knowledge, this is the first report showing profiles of FA transporter expression throughout the estrous cycle and during the peri-implantation period of early pregnancy in the uterine endometrium of livestock species. We revealed diverse expression of mRNA and/or protein of FA transporters in the porcine endometrium that was dependent on the reproductive status of animals, the day after estrus, and the isoform of FA transporter examined. Overall, CD36, SLC27A1, SLC27A4, and *SLC27A2* showed greater expression during pregnancy than during the estrous cycle. Accordingly, CCM and E2 used as conceptus signals stimulated FA transporter expression in the endometrium. Moreover, n-3 and n-6 PUFAs had mostly inhibitory effects on the expression of FA transporters in this tissue but also modulated the formation of PGE2 and PGI2 and the transcript abundance of genes encoding proteins crucial for endometrial development and function. These findings point to the existence of active transport of FAs in the porcine endometrium and the important roles of n-3 and n-6 PUFAs in the regulation of endometrial cell activity during the period of conceptus implantation.

Among reproductive organs, the importance of FA transporters is relatively well described for ovarian follicles [38] and the placenta [15,17]. Only a scarcity of data is available regarding uterine transporters of FAs. CD36 and SLC27A1 expression was examined in uterine fibroids [39] and endometrial carcinomas [40] of women. Moreover, results of global gene expression analyses of porcine [35,36,37] and bovine [41] endometrium showed that several genes related to lipid metabolism and action, including those encoding FA transporters, were among differentially expressed genes. Until now, however, there was no data describing how the expression of FA transporters changes during the estrous cycle and corresponding days of pregnancy and whether FAs may influence endometrial tissue. Results of the present study demonstrated dynamic changes in the expression of FA transporters in the endometrium primarily detected in pregnant animals. The mRNA expression of *SLC27A1*, *SLC27A2*, and *SLC27A4* increased in pregnant gilts between days 3–5 and 18–20, and on days 18–20, when formal implantation takes place, the abundance of all these transcripts was greater in pregnant than in cyclic animals. A similar increase was observed for *CD36* mRNA expression between days 9–10 and 18–20 of gestation. The greatest concentration of endometrial *SLC27A4* transcripts was detected on days 11–12 of pregnancy, the period corresponding to the maternal recognition of pregnancy in the pig. These results point to the role of FA transporters in conceptus implantation and further pregnancy progress in this species and are mostly consistent with the previous data of endometrial transcriptome examinations [35,36,37,42]. The microarray study revealed that concentrations of *SLC27A2* transcripts were greater in the porcine endometrium on day 24 compared with day 18 of pregnancy. Moreover, on both days of gestation, the mRNA expression of *SLC27A2* was greater than in day 13 cyclic gilts [37]. In confirmation, Zeng et al. [42] demonstrated that porcine luminal and glandular epithelium as well as stromal cells of the uterine endometrium express greater concentrations of *SLC27A2* transcripts on day 14 of pregnancy than on day 14 of the estrous cycle. Compared with cyclic gilts, *SLC27A1* mRNA expression in endometrial tissue of pregnant animals was lower on day 12 but greater on day 14 [35,36]. Moreover, *CD36* mRNA expression was elevated in the glandular epithelium of day 14 pregnant gilts as compared with cyclic animals [42]. All these results point to the modulatory role of conceptus presence in the uterine lumen on transcriptional activity of the porcine endometrium to facilitate and intensify FA transport during the peri-implantation period. Furthermore, profiles of *SLC27A1* and *SLC27A4* mRNA expression observed in the present study also indicate a possible regulation of these FA transporters by progesterone. Luteal synthesis of progesterone increases between days 1 and 12 after ovulation and is continued after day 14 of pregnancy but not the estrous cycle [43,44].

The present study showed that *SLC27A3* mRNA expression was lower during the period of the maternal recognition of pregnancy as compared with non-pregnant animals. Such a result is consistent with previous RNA sequencing data for porcine endometrium [36]. However, the role of SLC27A3 protein as an FA transporter is questionable as it primarily acts as an acyl-CoA ligase. Very little is known about the importance of SLC27A3 for cell function, but it is overexpressed in human lung and brain cancers [45]. *SLC27A6* mRNA expression, in turn, was greater in cyclic than in pregnant gilts on days 9–10 after estrus and decreased substantially thereafter regardless of the reproductive status of animals. Endometrial SLC27A6 protein content also decreased between days 9–10 and 18–20 after estrus. This indicates that in pigs, SLC27A6 may be important for the growth and development of the endometrium during the pre-receptive stage (before the maternal recognition of pregnancy) rather than during the period of conceptus implantation.

This is the first study examining both the tissue localization and expression profiles of FA transporters in the endometrium of domestic species. CD36, SLC27A1, SLC27A4, and SLC27A6 proteins were primarily localized in the luminal and glandular epithelium of the porcine endometrium. Strong positive staining of SLC27A1 and SLC27A6 was also detected in blood vessel walls, suggesting intense FA transport from the maternal circulation. Western blot results, in turn, showed that changes in FA transporter protein expression were not as dynamic as those observed for mRNA levels, but SLC27A1 and SLC27A4 protein expression increased between days 3–5 and 11–12 of pregnancy and on days 11–12 was greater in pregnant than in cyclic females. Similarly, endometrial CD36 protein levels tended to be elevated on days 11–12 of gestation as compared with respective days of the estrous cycle. These results support the mRNA data (present results) about the stimulatory effects of conceptus signals on FA transporter expression in the porcine endometrium during the period of the maternal recognition of pregnancy. On the other hand, greater mRNA expression observed in pregnant as compared with cyclic gilts on days 18–20 was not accompanied by elevated protein levels. Thus, some post-transcriptional regulations may exist to avoid excessive protein content. In contrast to the transcriptome, no proteomic data using endometrial tissue from the pig or other species are available to confirm or discuss the current results. Therefore, further studies are required to determine mechanisms controlling FA transporter expression in the uterus.

The observed differences in mRNA and/or protein levels between cyclic and pregnant gilts prompted us to examine the effect of the main conceptus signals, which were previously demonstrated as important regulators of the secretory activity of the porcine endometrium [33,34,46,47,48], on the expression of FA transporters. CCM that contained products secreted by in vitro incubated day 12 pig conceptuses increased CD36 protein level, stimulated *SLC27A1* mRNA expression, and tended to increase SLC27A1 protein content. Moreover, the incubation of endometrial explants with E2, known as the primary conceptus signal for the maternal recognition of pregnancy in the pig, resulted in greater concentrations of CD36 mRNA and protein as well as *SLC27A6* transcripts. By contrast, PGE2 inhibited *SLC27A4* mRNA expression. The current results indicate that conceptus products, including E2, which is secreted in great amount by elongating porcine conceptuses [33,48], may affect endometrial FA transporter expression.

The presence of FA transporters in the endometrium of pregnant gilts may be related to FA-affected changes in PG concentration and transcript abundance (as discussed below) but also to enhanced transport of FAs through the endometrium to support conceptus development. Global characterization of porcine intrauterine proteins during early gestation revealed that among identified proteins with increased concentration in the uterine lumen on day 13 (as compared with day 10) was salivary lipocalin [49]. This protein binds many lipids, including PUFAs, as demonstrated for the equine uterus and conceptus [50]. Interestingly, proteins involved in FA transport and metabolism were recently proposed as important for the elongation of porcine conceptuses [51].

Maternal diet influenced the expression of FA transporters in the mid-gestation mice placenta. FA transporters showed variable response patterns depending on the type of transporter examined and the day of pregnancy [52]. Moreover, prepartum cows fed with a diet supplemented with LA showed lower *CD36* and *FATP4* mRNA expression in placental tissue at term [23]. On the other hand, a positive correlation between *FATP4* mRNA expression and n-3 PUFAs percentage in maternal phospholipids was reported for the human term placenta [20]. Much less is known about the regulation of FA transporter expression in the porcine reproductive tissues. However, increasing the ratio of n-6 to n-3 PUFAs in feed resulted in greater *SLC27A1* and *SLC27A4* mRNA expression in subcutaneous adipose tissue in male pigs [53]. Results of the current in vitro study demonstrated that LA and ALA decreased *SLC27A1* mRNA expression, while LA and ARA down-regulated CD36 protein content in the porcine endometrium. Moreover, *SLC27A6* mRNA expression was lower after the addition of LA, DHA, or EPA into the incubation medium. By contrast, ARA, DHA, and EPA increased *SLC27A4* transcript abundance in the endometrial tissue. All these results (present data and cited studies) indicate diverse effects of PUFAs on the expression of their membrane transporters that may depend on the metabolic status of the tissue, PUFA composition in the diet, and the type of the transporter examined. Further studies should be undertaken to evaluate the consequences of the observed here mostly inhibitory effects of PUFAs on FA transporters.

For pigs, the majority of research is dedicated to examining the impact of PUFA supplementation during pregnancy on the reproductive parameters of the mother and offspring quality [54], and some positive effects of PUFAs on modern sows have been implicated [55]. Among various locally produced uterine factors, PGs are important modulators of both endometrial receptivity and conceptus development [33,34,56]. It is generally accepted that ARA released from membrane phospholipids is the main precursor for PG synthesis [57]. Feeding diets with a high proportion of n-6 PUFAs increased the plasma levels of PGF2α metabolite in dairy cows [58] and concentrations of PGE2 in the blood plasma of early pregnant gilts [59]. Furthermore, dietary supplementation of n-3 PUFAs in heifers [60] and sows [61] stimulated endometrial expression of PTGES, the terminal enzyme in PGE2 synthesis. As we demonstrated here, the treatment of endometrial explants with ARA resulted in elevated concentrations of PGE2 and 6-keto PGF1α (a stable metabolite of PGI2) in the incubation medium. Moreover, DHA and EPA increased PGE2 while DHA decreased PGI2 accumulation in the medium. This indicates a modulatory effect of both n-6 and n-3 PUFAs on PGE2 and PGI2 synthesis in the porcine endometrium, with a clearly visible greater impact of ARA on PGE2 output (14-fold vs. 2.6-fold increase for PGE2 and PGI2, respectively). However, none of the examined PUFAs affected PTGES or PTGIS protein expression, and some PUFAs showed even inhibitory effects on *PTGES* or *PTGIS* mRNA expression. This indicates that changes in PG levels observed in the current study result from changes in PTGES and PTGIS enzymatic activity rather than from changes in their concentrations. Similar observations were previously described for porcine endometrial tissue [62] and endometrial endothelial cells [63] exposed to cytokines, as well as for endometrial stromal cells exposed to conceptus signals [47]. Also, bovine myometrial cells responded to ARA with greater concentrations of PGE2 without differences in PTGES protein expression [64]. By contrast, EPA increased *PTGES* mRNA expression in cultured endometrial epithelial cells collected from cyclic ewes, which was not accompanied by increased PGE2 secretion [65]. This points to a complex regulation of PG synthesis. Nevertheless, results of the present study indicate that PUFAs of both the n-6 and n-3 series may serve as a source of substrates for PG synthesis in the endometrium of the pig, participating in endometrial receptivity as described for women [66,67].

In order to examine whether porcine endometrial cells may utilize FAs, selected genes related to FA binding, action, and metabolism were analyzed in the current study. FABPs are intracellular proteins involved in reversibly binding various FAs and trafficking them throughout cellular compartments, including mitochondria, endoplasmic reticulum, and nucleus [68]. Both porcine [37] and bovine [41] endometria of pregnant females showed greater concentrations of *FABP3* transcripts than their cyclic counterparts. Moreover, FABP4 has been shown as important for maintaining endometrial epithelial cell functions in humans [69] and embryo implantation in mice [70]. In the current study, *FABP3* mRNA expression in endometrial tissue was not affected by PUFA treatment. By contrast, LA and ALA stimulated *FABP4* mRNA expression, while LA, ARA, or EPA decreased *FABP5* mRNA expression. Our results are partly consistent with previous data showing that the treatment of human term placenta trophoblast cells with a mixture of LA and oleic acid had no effect on *FABP3* mRNA expression but increased both *FABP4* and *FABP5* transcript abundance [71]. Moreover, LA stimulated *FABP4* mRNA expression in human trophoblast cell lines [72]. Even so, FABP4 seems to be the main target of PUFAs that reach porcine endometrial cells.

Several FAs are ligands for PPARs, which function as transcription regulators [73]. PPARs have been described as mediators of lipid action on cellular metabolism and inflammatory reactions [73,74]. All three PPAR isoforms are present in porcine endometrial cells, and their activation modulates cell proliferation and the expression of genes involved in PG synthesis, nutrient transport, and angiogenesis [75]. Results of the current study showed that the mRNA expression of *PPARG*, but not *PPARA* or *PPARD*, was up-regulated by LA, ALA, DHA, and EPA in the porcine endometrium. Therefore, PUFA action in the porcine endometrium may be mediated by PPARG-dependent pathways.

Furthermore, we demonstrated that ARA stimulated the mRNA expression of *ACOX1* and *CPT1A,* genes that encode enzymes involved in FA oxidation in cells. Also, EPA increased *CPT1A* transcript abundance. Much greater expression of *ACOX1* has been previously demonstrated in the mouse uterus at implantation sites as compared with inter-implantation sites and was accompanied by the up-regulation of *SLC27A1* mRNA expression [76]. Increased *CPT1A* expression, in turn, along with *SLC27A6*, *PPARG*, and *FABP3/5* was demonstrated as crucial for porcine conceptus elongation [51]. In this study, greater expression of FA metabolism-related genes in the endometrium in response to ARA and EPA indicates a PUFA-dependent modulation of energy homeostasis processes that may be important for cellular remodeling of this tissue during the peri-implantation period in the pig.

PUFAs have been described as regulators of angiogenesis [72], and variable responses of n-3 and n-6 PUFAs were reported. In the human placenta, the pro-angiogenic action of n-3 PUFAs includes the stimulation of cell proliferation and tube formation, as well as the up-regulation of the expression of angiogenic factors [77,78]. Accordingly, increased mRNA expression of *VEGFA* and *FGF2* in endometrial tissue in response to ALA observed in the current research coincides with the previous data [77]. By contrast, no changes in angiogenic gene expression were observed in the presence of n-6 PUFAs, LA, or ARA. Therefore, PUFAs of the n-3 series seem to support pro-angiogenic gene expression in the porcine endometrium. However, more detailed research using endometrial endothelial cells should be undertaken to clarify the angiogenic activity of both n-3 and n-6 PUFAs in the porcine uterus.

Among various PUFAs examined in the present study, only ARA stimulated *IL1B* and inhibited *TNF* mRNA expression in the endometrial tissue. It is generally accepted that n-6 PUFAs exert pro-inflammatory activity in the organism, which is related to their role as precursors of various pro-inflammatory eicosanoids [78]. By contrast, PUFAs of the n-3 series are considered anti-inflammatory factors [74,79]. However, the final response may depend on individual FA, as a strong correlation was observed between several n-3 and n-6 FAs and pro-inflammatory cytokine concentrations in the blood plasma of pregnant women [80]. Based on the current results, we may conclude that ARA differentially modulates IL1β and TNFα synthesis in the endometrium of pregnant gilts, however, the exact mechanism should be further examined. Both cytokines, in turn, substantially modulate the secretory activity of the porcine uterus [81,82].

In the current study, some small (<2-fold) changes in mRNA or protein expression were observed. This may result from the heterogeneity of endometrial tissue. FA transporters were primarily localized in epithelial cells of the endometrium, but the final response of this tissue may be diluted by more numerous stromal cells. Moreover, less than 2-fold changes in transcript or protein levels detected in the porcine reproductive tissues still provided biologically meaningful insights, as shown using ‘-omic’ approaches [83,84,85].

In summary, this study is the first comprehensive approach to describe the expression and localization of FA transporters in the endometrium of pigs, accompanied by the examination of the possible role of FAs in this tissue. We demonstrated increased expression of CD36, SLC27A1, and SLC27A4 in the endometrium of pregnant compared with cyclic gilts and showed that conceptus products, including E2, may stimulate the expression of FA transporters. CD36, SLC27A1, SLC27A4, and SLC27A6 proteins were localized in the luminal and glandular epithelium of the uterine endometrium. Furthermore, we revealed that n-3 and n-6 PUFAs have modulatory effects on the expression of membrane FA transporters, intracellular factors involved in FA binding, action, and metabolism as well as on PGE2 and PGI2 secretion and the abundance of angiogenesis- and immune response-related transcripts. Taken together, n-6 and n-3 PUFAs seem to participate in endometrial preparation for conceptus implantation in the pig. Further research, however, should be undertaken to define cellular and molecular processes affected by PUFAs along with their consequences for pregnancy establishment and progress in the pig.

## 4. Materials and Methods

### 4.1. Animals and Sample Collection

All animals were subjected to commercial breeding procedures, and the samples were collected post-mortem during the regular slaughter process. Therefore, according to Directive 2010/63/EU of the European Parliament and of the Council of 22 September 2010 on the protection of animals used for scientific purposes, ethical review and approval were not required for this study. All experiments were conducted in accordance with the ARRIVE guidelines.

For ex vivo analyses of the expression of FA transporters in the endometrium, uteri were collected from 52 crossbred gilts (Polish Landrace × Duroc) of similar age, weight, and genetic background originating from one commercial farm. After exhibiting two consecutive estrous cycles, females were divided into two groups: cyclic and pregnant. Gilts assigned to the cyclic group were slaughtered on days 3–5 (*n* = 6), 9–10 (*n* = 5), 11–12 (*n* = 6), 15–16 (*n* = 5), and 18–20 (*n* = 5) of their third estrous cycle. The day of the estrous cycle was confirmed post-mortem by macroscopic observations of ovaries, as described earlier [86]. Gilts from the second group were bred 12 and 24 h after the detection of the estrus. The day of the second breeding was specified as day 1 of pregnancy. Animals were slaughtered on days 3–5 (*n* = 5), 9–10 (*n* = 5), 11–12 (*n* = 5), 15–16 (*n* = 5), and 18–20 (*n* = 5) of gestation. The day of pregnancy was verified by the morphology of conceptuses flushed from uterine horns (days 3 to 16) [87] or attached to the uterine wall (days 18–20) [88]. On days 3 to 16 of pregnancy, conceptuses were classified as follows: morula/blastocyst stage (days 3–5), spherical conceptuses with a diameter of less than 5 mm (days 9–10), all conceptuses filamentous in shape (days 11–12), all conceptuses elongated (days 15–16). On days 18–20 of pregnancy, embryos and trophoblast tissue with evident vascularization were distinguished. Endometrial tissue was separated from the myometrium, snap-frozen in liquid nitrogen, and stored at −80 °C for mRNA and protein expression analyses. For immunostaining procedures, several pieces of the uterus were fixed in 4% paraformaldehyde solution and embedded in paraffin. Moreover, kidney, liver, testis, and spleen samples were collected and used as positive control tissues for the presence of FA transporter proteins.

For in vitro experiments, endometrial tissue was collected from days 11–12 cyclic (*n* = 6; Experiment 1) and 15–16 pregnant (*n* = 5; Experiments 2 to 3) gilts (the same animals as described above). Moreover, day 12 conceptuses were used for the preparation of CCM (for Experiment 2).

A schematic presentation of the study is included in Supplementary Figure S8.

### 4.2. Experiment 1. Effect of Conceptus Signals on the Expression of FA Transporters in the Endometrium

CCM was prepared as described earlier [62], with modifications. Filamentous day 12 conceptuses were collected by gentle flushing of each uterine horn of pregnant females (*n* = 4) with Dulbecco’s Modified Eagle’s Medium/Ham’s Nutrient Mixture F-12 (DMEM/F-12; D2906; Sigma-Aldrich, St. Louis, MO, USA) containing antibiotics (10 IU/mL penicillin and 100 μg/mL streptomycin; P-4333; Sigma-Aldrich). Subsequently, conceptuses were weighed and placed in culture flasks in an appropriate volume (3 mL medium per 40 mg of conceptus tissue) of DMEM/F-12 supplemented with antibiotics and 0.1% bovine serum albumin (BSA; 81-003-3; Millipore, Kankakee, IL, USA). Incubation was carried out for 24 h at 37 °C in a humidified atmosphere of 5% CO2 and 95% air. The incubation medium was collected, centrifuged at 500× *g* for 5 min to remove tissue debris, and used as CCM to treat endometrial explants as described below.

To examine whether conceptus signals may affect the expression of FA transporters in the endometrium, 10 to 20 cm long fragments of the endometrial tissue were collected from the middle portion of a randomly selected uterine horn of days 11–12 cyclic gilts (*n* = 6), cut into small pieces (20–25 mg), placed into glass vials (5 explants per vial, a total of 100–110 mg tissue) containing 2 mL of the basal medium (DMEM/F-12 containing antibiotics and 0.1% BSA), and pre-incubated for 2 h. Subsequently, the medium was discarded and endometrial explants were treated with the basal medium, CCM mixed 3:1 with the basal medium, or the basal medium containing E2 (E2758; Sigma-Aldrich; 100 nM), PGE2 (14010; Cayman Chemical, Ann Arbor, MI, USA; 100 nM), IL1β (I9401; Sigma-Aldrich; 20 ng/mL), or interferon γ (IFNγ; PCS4034; BioSource International Inc., Camarillo, CA, USA; 20 ng/mL) for the next 24 h at 37 °C in a humidified atmosphere of 5% CO2 and 95% air. All treatments were performed in duplicate in six separate experiments (gilts). After incubation, endometrial explants were washed with sterile PBS, snap-frozen in liquid nitrogen, and stored at −80 °C for further analyses of CD36, SLC27A1, SLC27A4, and SLC27A6 expression.

### 4.3. Experiment 2. Effect of n-6 and n-3 PUFAs on the Expression of FA Transporters in the Endometrium

To examine whether PUFAs may affect the expression of FA transporters in the endometrium, uteri from days 15–16 pregnant gilts (*n* = 5) were used for the preparation of endometrial explants as described above for Experiment 1. Pre-incubation of endometrial explants was conducted for 2 h in the basal medium (Dulbecco’s Modified Eagle’s Medium [DMEM; D2902; Sigma-Aldrich] containing 15 mM HEPES [H4034; Sigma-Aldrich], antibiotics and 1% FA-free BSA [03117057001; Roche Diagnostics GmbH, Mannheim, Germany]). Next, the medium was removed and endometrial explants were exposed to the basal medium or the basal medium containing one of the following PUFAs (all from Cayman Chemical): linoleic acid (LA; 90150), arachidonic acid (ARA; 90010), α-linolenic acid (ALA; 90210), docosahexaenoic acid (DHA; 90310), or eicosapentaenoic acid (EPA; 21908) used at the concentration of 200 μM. PUFAs were prepared as described previously [22]. Incubation was conducted for 24 h at 37 °C in a humidified atmosphere of 5% CO2 and 95% air. All treatments were performed in duplicate in five separate experiments (gilts). After incubation, endometrial explants were washed with sterile PBS, snap-frozen in liquid nitrogen, and stored at −80 °C for further analyses of CD36, SLC27A1, SLC27A4, and SLC27A6 expression.

### 4.4. Experiment 3. Effect of n-6 and n-3 PUFAs on PGE2 and PGI2 Synthesis and the Abundance of Selected Transcripts in the Endometrium

To examine whether PUFAs may influence endometrial activity during early pregnancy, uteri from days 15–16 pregnant gilts (*n* = 5) were collected and endometrial explants were prepared and treated with LA, ARA, ALA, DHA, or EPA as described for Experiment 2. All treatments were performed in duplicate in five separate experiments (gilts). After incubation, tissue explants were washed with sterile PBS, snap-frozen in liquid nitrogen, and stored at −80 °C to determine the mRNA and protein expression of enzymes involved in PGE2 and PGI2 synthesis and the abundance of transcripts encoding factors involved in intracellular FA binding (*FABP3*, *FABP4*, *FABP5*), action (*PPARA*, *PPARD*, *PPARG*), or metabolism (*ACOX1*, *CPT1A*) as well as in angiogenesis (*VEGFA*, *FGF2*) and immune response (*IL1B*, *IL6*, *TNF*). Moreover, incubation media were collected and stored at −40 °C until analyses of PGE2 and PGI2 concentrations.

### 4.5. Total RNA Isolation and Real-Time PCR

Total RNA was extracted using a Total RNA Mini kit (031-100; A&A Biotechnology, Gdansk, Poland) and treated with DNase I (AMPD1; Sigma-Aldrich) in accordance with the manufacturer’s instructions. Reverse transcription of RNA samples (1 μg) was performed using a High Capacity Reverse Transcription Kit (4374966; Applied Biosystems by Thermo Fisher Scientific; Waltman, MA, USA) as described earlier [62].

Diluted cDNA from RT-PCR was used to determine the relative mRNA abundance of selected genes with an ABI Viia7 Sequence Detection System (Life Technologies Inc., Carlsbad, CA, USA). To evaluate *CD36*, *SLC27A1*, *SLC27A2*, *SLC27A3*, *SLC27A4*, *SLC27A6*, *PTGES*, *PTGIS*, *FABP3*, *FABP4*, *FABP5*, *PPARA*, *PPARD*, *PPARG*, *ACOX1*, *CPT1A*, *VEGFA*, *FGF2*, *IL1B*, *IL6*, *TNF*, *HPRT1*, *GAPDH*, and *ACTG1* gene expression, 15 ng of complementary cDNA was amplified using TaqMan Gene Expression assays (Applied Biosystems by Thermo Fisher Scientific). All abbreviations of the examined genes, their full names, and the ID numbers of TaqMan probes are listed in Supplementary Table S1. Each PCR reaction was performed in duplicates in a 384-well plate under the following conditions: initial denaturation for 10 min at 95 °C, followed by 40 cycles of 15 s denaturation at 95 °C and 60 s of annealing at 60 °C. To check for genomic DNA contamination, the control reactions in the absence of reverse transcriptase were performed. Moreover, no template controls with nuclease-free water were conducted to test for possible reagent contamination. Data from real-time PCR were analyzed using the PCR Miner algorithm [89]. The NormFinder software version 0953 [90] was applied to select the most stable reference genes among *GAPDH*, *HPRT1*, and *ACTG1*. All expression data for each target gene were normalized against geometric averaging of *HPRT1* and *GAPDH.*

### 4.6. Western Blot Analysis

Endometrial and control tissues were homogenized using an ice-cold homogenization buffer (50 mM Tris-HCl, pH 8.0; 150 mM NaCl, 1% Triton X-100, 1 mM EDTA) containing protease inhibitor cocktail (P8340; Sigma-Aldrich) in Lysing Matrix D (MP Biomedicals, Solon, OH, USA) with a FastPrep-24 instrument (MP Biomedicals) for the endometrial tissue or with a POLYTRON^®^ PT 1200 E (INTER-CHEM, Poznan, Poland) for endometrial explants. Homogenates were centrifuged for 10 min at 800× *g* at 4 °C. Supernatants were collected and stored for western blot analysis.

Total protein extracts of endometrial tissue (8 µg for SLC27A1 and SLC27A4; 20 µg for CD36 and SLC27A6) or endometrial explants (10 µg for SLC27A1, SLC27A4, and PTGES; 15 µg for PTGIS; 20 µg for CD36 and SLC27A6) were dissolved in SDS gel-loading buffer (50 mM Tris-HCl, pH 6.8; 4% SDS, 20% glycerol, and 2% β-mercaptoethanol), heated to 95 °C for 5 min, and separated on 10% (CD36, SLC27A1, SLC27A4, SLC27A6, and PTGIS) or 12% (PTGES) SDS-PAGE. Separated proteins were electroblotted onto 0.45-µm pore size polyvinylidene difluoride membrane in a transfer buffer (20 mM Tris-HCl, pH 8.2; 150 mM glycine, 20% methanol [*v*/*v*]). The nonspecific binding sites were blocked with 5% nonfat dry milk with TBS-T (Tris-buffered saline, containing 0.1% Tween-20) for 1.5 h at room temperature. Subsequently, the membranes were incubated overnight with an appropriate primary antibody (Supplementary Table S2) at 4 °C, washed with TBS-T, and incubated for 1 h with anti-rabbit IgG (whole molecule), alkaline phosphatase antibody (A3687; Sigma-Aldrich; 1:20,000), or Immun-Star^TM^ goat anti-rabbit (GAR)-HRP conjugate (170-5046; Bio-Rad Laboratories, Inc., Hercules, CA, USA; 1:20,000) depending on further visualization procedure. For CD36 detection, immune complexes were visualized using a standard alkaline phosphatase visualization procedure. For SLC27A1, SLC27A4, SLC27A6, PTGES, and PTGIS detection, immune complexes were visualized using a Clarity Western ECL Substrate kit (Bio-Rad Laboratories). Images were captured with the ChemiDoc^TM^ Touch Imaging System and quantified using Image Lab 6 software (both from Bio-Rad Laboratories). An internal control for protein loading was performed by re-blocking membranes with 5% nonfat dry milk and further incubation with anti-ACTB or anti-GAPDH antibodies (Supplementary Table S2).

### 4.7. Immunostaining of the Uterine Endometrium

FA transporter proteins were localized using IF (for CD36) or IHC (for SLC27A1, SLC27A4, and SLC27A6) procedures. Paraffin-embedded fragments of the endometrium, spleen, and kidney were cut into 5 μm sections and mounted on SuperFrost Plus microscope slides (Menzel-Gläzer; Braunschweig, Germany). Paraffin was removed by heating slices at 58 °C and washing in xylene. After rehydration in a graded series of ethanol (100 to 50%), the antigen retrieval was conducted by heating slides in citrate buffer (10 mM sodium citrate, 0.05% Tween 20; pH 6.0) for 15 min. Afterwards, 30% hydrogen peroxidase in methanol was added for 15 min followed by 1 h treatment of slides with Fish Serum Blocking Buffer (37527; Thermo Fisher Scientific). Then, an overnight incubation with primary antibodies (listed in Supplementary Table S2) was performed at 4 °C. The next day, slides were treated depending on the procedure applied to visualize FA transporter proteins. For IF (CD36), sections were incubated for 1 h in the dark with Alexa Fluor^TM^ 594 donkey anti-rabbit IgG (A21207; Invitrogen by Thermo Fisher Scientific; 1:5000), washed with TBS, and mounted in VECTASHIELD Mounting Medium (Vector Laboratories, Inc., Burlingame, CA, USA) containing diamidino-2-phenylindole (DAPI) to stain nuclei. For IHC (SLC27A1, SLC27A4, and SLC27A6), sections were incubated for 30 min with a goat anti-rabbit IgG secondary antibody (BA-1000; Vector Laboratories; 1:2000), washed with TBS, and treated with a mixture of Reagents A and B from VECTASTAIN^®^ Elite^®^ ABC-HRP kit, Peroxidase Rabbit IgG (PK-6101; Vector Laboratories). Subsequently, slides were treated with 3,3′-diamidinobenzidine (D5637; Sigma-Aldrich), counterstained with hematoxylin, dehydrated, and mounted using DPX (06522; Sigma-Aldrich). Negative controls were accomplished by replacing primary antibodies with a rabbit IgG negative control (I-1000, Vector Laboratories) according to the manufacturer’s instructions. Slides were photographed using Zeiss AXIO Imager.Z1 microscope (Carl Zeiss Microscopy GmbH, Jena, Germany).

### 4.8. Immunoassay

To examine concentrations of PGE2 in the incubation medium, a direct EIA method [91] was used. Anti-PGE2 antibody (P-5164; Sigma-Aldrich) developed in rabbits was applied at the dilution of 1:200. The sensitivity of the assay was 0.19 ng/mL, and the intra- assay coefficient of variation was 11.8%. Levels of PGE2 in the medium were standardized per wet weight of endometrial explants.

To determine concentrations of PGI2 metabolite in the incubation medium, a 6-keto PGF1α ELISA kit (515211; Cayman Chemical) was used according to the manufacturer’s instructions. The sensitivity of the assay was 1.6 pg/mL, and the intra-assay coefficient of variation was 9.4%. Levels of 6-keto PGF1α in the media were standardized per wet weight of endometrial explants.

### 4.9. Statistical Analyses

All statistical analyses were performed using GraphPad PRISM v. 10 (GraphPad Software, Inc., San Diego, CA, USA). To analyze profiles of mRNA and protein expression of FA transporters in the endometrium, two-way ANOVA followed by the Bonferroni post hoc test was used. This analysis included the effect of the day after estrus, reproductive status (cyclic vs. pregnant) of gilts, and day by reproductive status interaction. To test (1) the effect of conceptus signals on FA transporter expression and the effect of PUFAs on (2) FA transporter expression, (3) PGE2 and PGI2 synthesis, and (4) the abundance of selected transcripts in endometrial explants, one-way ANOVA followed by Dunnett’s post hoc test was performed. All numerical data are presented as means ± SEM, and means were considered to be statistically different at *p* ≤ 0.05 with a tendency estimated at 0.07 ≥ *p* > 0.05.

## Data Availability

The raw data supporting the conclusions of this article will be made available by the authors on reasonable request.

## Supplementary Materials

The following supporting information can be downloaded at: https://www.mdpi.com/article/10.3390/ijms252011102/s1.

## References

[1] Gaskins A.J., Chavarro J.E. (2018). Diet and fertility: A review. Am. J. Obstet. Gynecol..

[2] Silvestris E., Lovero D., Palmirotta R. (2019). Nutrition and Female Fertility: An Interdependent Correlation. Front. Endocrinol..

[3] Koga F., Kitagami S., Izumi A., Uemura T., Takayama O., Koga T., Mizoguchi T. (2020). Relationship between nutrition and reproduction. Reprod. Med. Biol..

[4] Skoracka K., Ratajczak A.E., Rychter A.M., Dobrowolska A., Krela-Kaźmierczak I. (2021). Female Fertility and the Nutritional Approach: The Most Essential Aspects. Adv. Nutr..

[5] Casares D., Escribá P.V., Rosselló C.A. (2019). Membrane Lipid Composition: Effect on Membrane and Organelle Structure, Function and Compartmentalization and Therapeutic Avenues. Int. J. Mol. Sci..

[6] Herrera E. (2002). Implications of Dietary Fatty Acids During Pregnancy on Placental, Fetal and Postnatal Development—A Review. Placenta.

[7] Jump D.B. (2004). Fatty Acid Regulation of Gene Transcription. Crit. Rev. Clin. Lab. Sci..

[8] Nakamura M.T., Cheon Y., Li Y., Nara T.Y. (2004). Mechanisms of regulation of gene expression by fatty acids. Lipids.

[9] Calder P.C. (2012). Long-chain fatty acids and inflammation. Proc. Nutr. Soc..

[10] Georgiadi A., Kersten S. (2012). Mechanisms of Gene Regulation by Fatty Acids. Adv. Nutr..

[11] Basak S., Duttaroy A.K. (2023). Maternal PUFAs, Placental Epigenetics, and Their Relevance to Fetal Growth and Brain Development. Reprod. Sci..

[12] Saini R.K., Keum Y.-S. (2018). Omega-3 and omega-6 polyunsaturated fatty acids: Dietary sources, metabolism, and significance—A review. Life Sci..

[13] Duttaroy A.K., Basak S. (2020). Maternal dietary fatty acids and their roles in human placental development. Prostaglandins, Leukot. Essent. Fatty Acids.

[14] Kamp F., Hamilton J.A. (2006). How fatty acids of different chain length enter and leave cells by free diffusion. Prostaglandins, Leukot. Essent. Fatty Acids.

[15] Larqué E., Demmelmair H., Gil-Sánchez A., Prieto-Sánchez M.T., Blanco J.E., Pagán A., Faber F.L., Zamora S., Parrilla J.J., Koletzko B. (2011). Placental transfer of fatty acids and fetal implications. Am. J. Clin. Nutr..

[16] Glatz J.F.C., Luiken J.J.F.P., Bonen A. (2010). Membrane Fatty Acid Transporters as Regulators of Lipid Metabolism: Implications for Metabolic Disease. Physiol. Rev..

[17] Duttaroy A.K. (2009). Transport of fatty acids across the human placenta: A review. Prog. Lipid Res..

[18] Jones M.L., Mark P.J., Waddell B.J. (2014). Maternal dietary omega-3 fatty acids and placental function. Reproduction.

[19] Campbell F.M., Bush P.G., Veerkamp J.H., Dutta-Roy A.K. (1998). Detection and cellular localization of plasma membrane-associated and cytoplasmic fatty acid-binding proteins in human placenta. Placenta.

[20] Larqué E., Krauss-Etschmann S., Campoy C., Hartl D., Linde J., Klingler M., Demmelmair H., Caño A., Gil A., Bondy B. (2006). Docosahexaenoic acid supply in pregnancy affects placental expression of fatty acid transport proteins. Am. J. Clin. Nutr..

[21] Basak S., Duttaroy A.K. (2013). Effects of fatty acids on angiogenic activity in the placental extravillious trophoblast cells. Prostaglandins Leukot. Essent. Fatty Acids.

[22] Leroy C., Tobin K.A.R., Basak S., Staff A.C., Duttaroy A.K. (2017). Fatty acid-binding protein3 expression in BeWo cells, a human placental choriocarcinoma cell line. Prostaglandins Leukot. Essent. Fatty Acids.

[23] Salehi R., Ambrose D.J. (2017). Prepartum maternal diets supplemented with oilseeds alter the fatty acid profile in bovine neonatal plasma possibly through reduced placental expression of fatty acid transporter protein 4 and fatty acid translocase. Reprod. Fertil. Dev..

[24] Steinhauser C.B., Askelson K., Lambo C.A., Hobbs K.C., Bazer F.W., Satterfield M.C. (2021). Lipid metabolism is altered in maternal, placental, and fetal tissues of ewes with small for gestational age fetuses†. Biol. Reprod..

[25] McNeel A.K., Chen C., Schroeder S., Sonstegard T., Dawson H., Vallet J.L. (2019). Application of RNA-seq transcriptomic analysis to reproductive physiology of the pig: Insights into differential trophoblast function within the late gestation porcine placenta. Control. Pig Reprod. IX.

[26] Tian L., Dong S.S., Hu J., Yao J.J., Yan P.S. (2018). The effect of maternal obesity on fatty acid transporter expression and lipid metabolism in the full-term placenta of lean breed swine. J. Anim. Physiol. Anim. Nutr..

[27] Ao Z., Wu X., Zhou J., Gu T., Wang X., Shi J., Zhao C., Cai G., Zheng E., Liu D. (2019). Cloned pig fetuses exhibit fatty acid deficiency from impaired placental transport. Mol. Reprod. Dev..

[28] Achache H., Revel A. (2006). Endometrial receptivity markers, the journey to successful embryo implantation. Hum. Reprod. Update.

[29] Bazer F.W., Spencer T.E., Johnson G.A., Burghardt R.C. (2011). Uterine receptivity to implantation of blastocysts in mammals. Front. Biosci..

[30] Garratt J., Rahmati M. (2023). Assessing the endometrium: An update on current and potential novel biomarkers of receptivity. J. Reprod. Immunol..

[31] Spencer T.E., Johnson G.A., Burghardt R.C., Bazer F.W. (2004). Progesterone and Placental Hormone Actions on the Uterus: Insights from Domestic Animals. Biol. Reprod..

[32] Ziecik A.J., Waclawik A., Kaczmarek M.M., Blitek A., Jalali B.M., Andronowska A. (2011). Mechanisms for the Establishment of Pregnancy in the Pig. Reprod. Domest. Anim..

[33] Bazer F.W., Johnson G.A. (2014). Pig blastocyst–uterine interactions. Differentiation.

[34] Waclawik A., Kaczmarek M.M., Blitek A., Kaczynski P., Ziecik A.J. (2017). Embryo-maternal dialogue during pregnancy establishment and implantation in the pig. Mol. Reprod. Dev..

[35] Samborski A., Graf A., Krebs S., Kessler B., Bauersachs S. (2013). Deep Sequencing of the Porcine Endometrial Transcriptome on Day 14 of Pregnancy. Biol. Reprod..

[36] Samborski A., Graf A., Krebs S., Kessler B., Reichenbach M., Reichenbach H.-D., Ulbrich S.E., Bauersachs S. (2013). Transcriptome Changes in the Porcine Endometrium During the Preattachment Phase. Biol. Reprod..

[37] Chen X., Li A., Chen W., Wei J., Fu J., Wang A. (2015). Differential Gene Expression in Uterine Endometrium During Implantation in Pigs. Biol. Reprod..

[38] Uzbekova S., Bertevello P.S., Dalbies-Tran R., Elis S., Labas V., Monget P., Teixeira-Gomes A.-P. (2021). Metabolic exchanges between the oocyte and its environment: Focus on lipids. Reprod. Fertil. Dev..

[39] Knapp P., Chabowski A., Posmyk R., Górski J. (2016). Expression of the energy substrate transporters in uterine fibroids. Prostaglandins Other Lipid Mediat..

[40] Knapp P., Chabowski A., Harasiuk D., Górski J. (2012). Reversed Glucose and Fatty Acids Transporter Expression in Human Endometrial Cancer. Horm. Metab. Res..

[41] Forde N., Carter F., Spencer T.E., Bazer F.W., Sandra O., Mansouri-Attia N., Okumu L.A., McGettigan P.A., Mehta J.P., McBride R. (2011). Conceptus-Induced Changes in the Endometrial Transcriptome: How Soon Does the Cow Know She Is Pregnant?. Biol. Reprod..

[42] Zeng S., Ulbrich S.E., Bauersachs S. (2019). Spatial organization of endometrial gene expression at the onset of embryo attachment in pigs. BMC Genom..

[43] Masuda H., Anderson L.L., Henricks D.M. (1967). Progesterone in Ovarian Venous Plasma and Corpora Lutea of the Pig1. Endocrinology.

[44] Ziecik A.J., Przygrodzka E., Kaczmarek M.M. (2017). Corpus Luteum Regression and Early Pregnancy Maintenance in Pigs. The Life Cycle of the Corpus Luteum.

[45] Acharya R., Shetty S.S., Kumari N S. (2023). Fatty acid transport proteins (FATPs) in cancer. Chem. Phys. Lipids.

[46] Johnson G.A., Bazer F.W., Burghardt R.C., Spencer T.E., Wu G., Bayless K.J. (2009). Conceptus-uterus interactions in pigs: Endometrial gene expression in response to estrogens and interferons from conceptuses. Soc. Reprod. Fertiil Suppl..

[47] Blitek A., Morawska E., Kiewisz J., Ziecik A.J. (2011). Effect of conceptus secretions on HOXA10 and PTGS2 gene expression, and PGE2 release in co-cultured luminal epithelial and stromal cells of the porcine endometrium at the time of early implantation. Theriogenology.

[48] Geisert R.D., Johnson G.A., Burghardt R.C. (2015). Implantation and Establishment of Pregnancy in the Pig. Adv. Anat. Embryol. Cell. Biol..

[49] Kayser J.-P.R., Kim J.G., Cerny R.L., Vallet J.L. (2006). Global characterization of porcine intrauterine proteins during early pregnancy. Reproduction.

[50] Stewart F., Kennedy M.W., Suire S. (2000). A novel uterine lipocalin supporting pregnancy in equids. Cell. Mol. Life Sci..

[51] Miles J.R., Walsh S.C., Rempel L.A., Pannier A.K. (2023). Mechanisms regulating the initiation of porcine conceptus elongation. Mol. Reprod. Dev..

[52] Kappen C., Kruger C., Jones S., Herion N.J., Salbaum J.M. (2019). Maternal diet modulates placental nutrient transporter gene expression in a mouse model of diabetic pregnancy. PLoS ONE.

[53] Li F., Duan Y., Li Y., Tang Y., Geng M., Oladele O.A., Kim S.W., Yin Y. (2015). Effects of dietary *n*-6:*n*-3 PUFA ratio on fatty acid composition, free amino acid profile and gene expression of transporters in finishing pigs. Br. J. Nutr..

[54] Tanghe S., De Smet S. (2013). Does sow reproduction and piglet performance benefit from the addition of n-3 polyunsaturated fatty acids to the maternal diet?. Vet. J..

[55] Rosero D.S., Boyd R.D., McCulley M., Odle J., van Heugten E. (2016). Essential fatty acid supplementation during lactation is required to maximize the subsequent reproductive performance of the modern sow. Anim. Reprod. Sci..

[56] Kennedy T.G., Gillio-Meina C., Phang S.H. (2007). Prostaglandins and the initiation of blastocyst implantation and decidualization. Reproduction.

[57] Smith W.L., Garavito R.M., DeWitt D.L. (1996). Prostaglandin Endoperoxide H Synthases (Cyclooxygenases)-1 and -2. J. Biol. Chem..

[58] Petit H.V., Germiquet C., Lebel D. (2004). Effect of Feeding Whole, Unprocessed Sunflower Seeds and Flaxseed on Milk Production, Milk Composition, and Prostaglandin Secretion in Dairy Cows. J. Dairy Sci..

[59] Chartrand R., Matte J.J., Lessard M., Chouinard P.Y., Giguère A., Laforest J.P. (2003). Effect of dietary fat sources on systemic and intrauterine synthesis of prostaglandins during early pregnancy in gilts. J. Anim. Sci..

[60] Coyne G.S., Kenny D.A., Childs S., Sreenan J.M., Waters S.M. (2008). Dietary n-3 polyunsaturated fatty acids alter the expression of genes involved in prostaglandin biosynthesis in the bovine uterus. Theriogenology.

[61] Gokuldas P.P., Singh S.K., Tamuli M.K., Naskar S., Vashi Y., Thomas R., Barman K., Pegu S.R., Chethan S.G., Agarwal S.K. (2018). Dietary supplementation of n-3 polyunsaturated fatty acid alters endometrial expression of genes involved in prostaglandin biosynthetic pathway in breeding sows (Sus scrofa). Theriogenology.

[62] Morawska E., Kaczmarek M.M., Blitek A. (2012). Regulation of prostacyclin synthase expression and prostacyclin content in the pig endometrium. Theriogenology.

[63] Szymanska M., Blitek A. (2023). Diverse effects of prostacyclin on angiogenesis-related processes in the porcine endometrium. Sci. Rep..

[64] Slonina D., Kowalik M.K., Subocz M., Kotwica J. (2009). The effect of ovarian steroids on oxytocin-stimulated secretion and synthesis of prostaglandins in bovine myometrial cells. Prostaglandins Other Lipid Mediat..

[65] Cheng Z., Abayasekara D.R.E., Ward F., Preece D.M., Raheem K.A., Wathes D.C. (2013). Altering n-3 to n-6 polyunsaturated fatty acid ratios affects prostaglandin production by ovine uterine endometrium. Anim. Reprod. Sci..

[66] Yang T., Zhao J., Liu F., Li Y. (2022). Lipid metabolism and endometrial receptivity. Hum. Reprod. Update.

[67] Chen M., Zheng Z., Shi J., Shao J. (2021). Insight on Polyunsaturated Fatty Acids in Endometrial Receptivity. Biomolecules.

[68] Smathers R.L., Petersen D.R. (2011). The human fatty acid-binding protein family: Evolutionary divergences and functions. Hum. Genom..

[69] Zhu Q., Jin Y., Wang P., Wang H., Lu B., Wang Z., Dong M. (2015). Expression and function of fatty acid-binding protein 4 in epithelial cell of uterine endometrium. Cell Biol. Int..

[70] Wang P., Zhu Q., Peng H., Du M., Dong M., Wang H. (2017). Fatty Acid-Binding Protein 4 in Endometrial Epithelium Is Involved in Embryonic Implantation. Cell. Physiol. Biochem..

[71] Scifres C.M., Chen B., Nelson D.M., Sadovsky Y. (2011). Fatty Acid Binding Protein 4 Regulates Intracellular Lipid Accumulation in Human Trophoblasts. J. Clin. Endocrinol. Metab..

[72] Basak S., Das M.K., Duttaroy A.K. (2013). Fatty acid-induced angiogenesis in first trimester placental trophoblast cells: Possible roles of cellular fatty acid-binding proteins. Life Sci..

[73] Varga T., Czimmerer Z., Nagy L. (2011). PPARs are a unique set of fatty acid regulated transcription factors controlling both lipid metabolism and inflammation. Biochim. Biophys. Acta.

[74] Wu D. (2004). Modulation of immune and inflammatory responses by dietary lipids. Curr. Opin. Lipidol..

[75] Blitek A., Szymanska M. (2019). Regulation of expression and role of peroxisome proliferator-activated receptors (PPARs) in luminal epithelial and stromal cells of the porcine endometrium. Theriogenology.

[76] Kim S.T., Marquard K., Stephens S., Louden E., Allsworth J., Moley K.H. (2011). Adiponectin and adiponectin receptors in the mouse preimplantation embryo and uterus. Hum. Reprod..

[77] Johnsen G.M., Basak S., Weedon-Fekjær M.S., Staff A.C., Duttaroy A.K. (2011). Docosahexaenoic acid stimulates tube formation in first trimester trophoblast cells, HTR8/SVneo. Placenta.

[78] Godhamgaonkar A.A., Wadhwani N.S., Joshi S.R. (2020). Exploring the role of LC-PUFA metabolism in pregnancy complications. Prostaglandins Leukot. Essent. Fatty Acids.

[79] Joshi N.P., Madiwale S.D., Sundrani D.P., Joshi S.R. (2023). Fatty acids, inflammation and angiogenesis in women with gestational diabetes mellitus. Biochimie.

[80] Chen X., Stein T.P., Steer R.A., Scholl T.O. (2019). Individual free fatty acids have unique associations with inflammatory biomarkers, insulin resistance and insulin secretion in healthy and gestational diabetic pregnant women. BMJ Open Diabetes Res. Care.

[81] Blitek A., Ziecik A.J. (2006). Role of Tumour Necrosis Factor *α* in Stimulation of Prostaglandins F_2*α*_ and E_2_ Release by Cultured Porcine Endometrial Cells. Reprod. Domest. Anim..

[82] Franczak A., Zmijewska A., Kurowicka B., Wojciechowicz B., Kotwica G. (2010). Interleukin 1β-induced synthesis and secretion of prostaglandin E₂ in the porcine uterus during various periods of pregnancy and the estrous cycle. J. Physiol. Pharmacol..

[83] Kiewisz J., Krawczyński K., Lisowski P., Blitek A., Zwierzchowski L., Ziecik A.J., Kaczmarek M.M. (2014). Global gene expression profiling of porcine endometria on Days 12 and 16 of the estrous cycle and pregnancy. Theriogenology.

[84] Likszo P., Skarzynski D.J., Jalali B.M. (2021). Changes in Porcine Corpus Luteum Proteome Associated with Development, Maintenance, Regression, and Rescue during Estrous Cycle and Early Pregnancy. Int. J. Mol. Sci..

[85] Szuszkiewicz J., Myszczynski K., Reliszko Z.P., Heifetz Y., Kaczmarek M.M. (2022). Early steps of embryo implantation are regulated by exchange of extracellular vesicles between the embryo and the endometrium. FASEB J..

[86] Akins E.L., Morrissette M.C. (1968). Gross ovarian changes during estrous cycle of swine. Am. J. Vet. Res..

[87] Anderson L.L. (1978). Growth, protein content and distribution of early pig embryos. Anat. Rec..

[88] Vallet J.L., Miles J.R., Freking B.A. (2009). Development of the pig placenta. Soc. Reprod. Fertil. Suppl..

[89] Zhao S., Fernald R.D. (2005). Comprehensive Algorithm for Quantitative Real-Time Polymerase Chain Reaction. J. Comput. Biol..

[90] Andersen C.L., Jensen J.L., Ørntoft T.F. (2004). Normalization of Real-Time Quantitative Reverse Transcription-PCR Data: A Model-Based Variance Estimation Approach to Identify Genes Suited for Normalization, Applied to Bladder and Colon Cancer Data Sets. Cancer Res..

[91] Blitek A., Waclawik A., Kaczmarek M.M., Kiewisz J., Ziecik A.J. (2010). Effect of estrus induction on prostaglandin content and prostaglandin synthesis enzyme expression in the uterus of early pregnant pigs. Theriogenology.

