# Supplementary material for: Expression Profiles of Fatty Acid Transporters and the Role of n-3 and n-6 Polyunsaturated Fatty Acids in the Porcine Endometrium
Source: Int J Mol Sci. 2024 Oct 16;25(20):11102. doi: 10.3390/ijms252011102 (PMC11507490; doi:10.3390/ijms252011102)
Supplement: Supplementary file 1 [file ijms-25-11102-s001.zip › Blitek_and_Szymanska_IJMS_Supplementary_Figures.pdf]

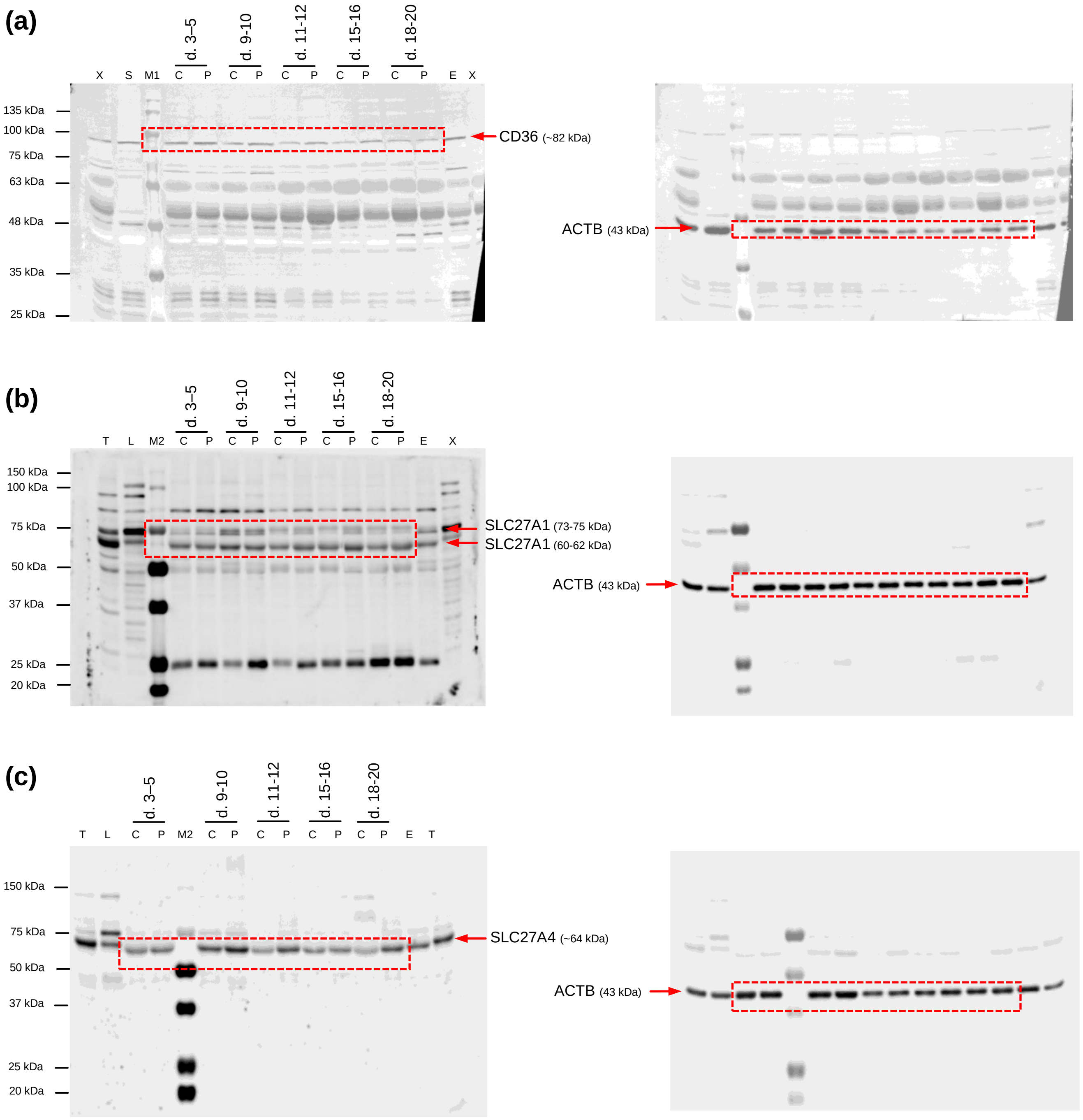

Supplementary Fig. S1. Western blot results presenting CD36 (**a**), SLC27A1 (**b**) and SLC27A4 (**c**) protein expression in endometrial tissue of cyclic and pregnant gilts (left panel). β-actin (ACTB; right panel) was used as an internal control of protein loading. After being photographed, blots presented in the left panel were re-probed for ACTB and photographed again. Spleen (S) was used as a positive control for CD36 protein expression. Testis (T) and liver (L) were used as positive controls for SLC27A1 and SLC27A4 protein expression. M1: marker (E3210-01, EURx, Gdansk, Poland); M2: marker (161-0373, Bio-Rad Laboratories, Inc., Hercules, USA); d.: day after estrus; C: cyclic; P: pregnant; E: endometrial tissue (the reference sample applied in each blot); X: unrelated samples (randomly selected samples applied in marginal paths to avoid excessive bending of bands at the edge of gels). The red dashed lines show the fragment of each blot presented in Figure 2.

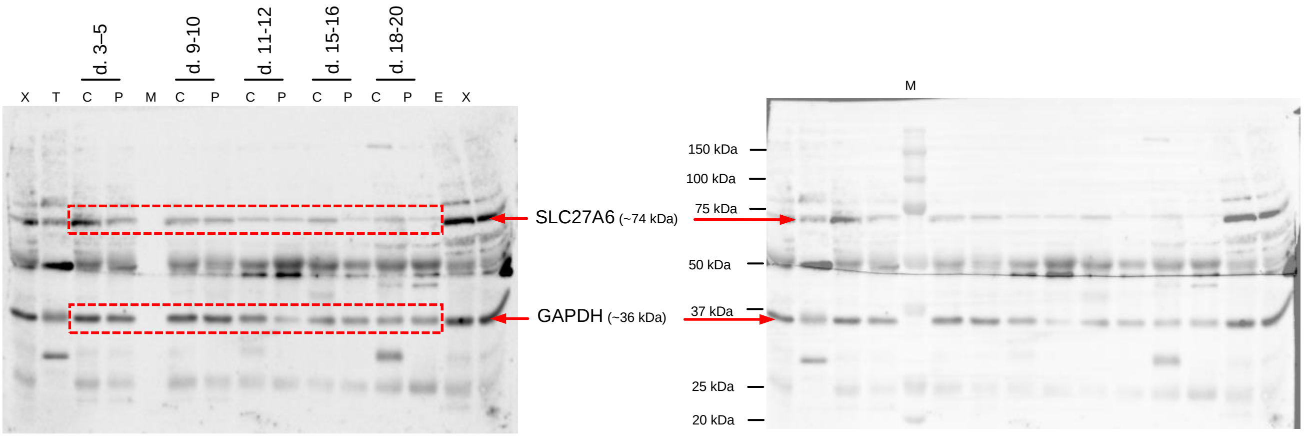

Supplementary Fig. S2. Western blot results presenting SLC27A6 protein expression in endometrial tissue of cyclic and pregnant gilts (left panel). Glyceraldehyde-3-phosphate dehydrogenase (GAPDH) was used as an internal control of protein loading. After transfer, PVDF membrane was cut at about 50 kDa and the upper part was incubated with anti-SLC27A6 antibody while the lower part was incubated with an anti-GAPDH antibody. Testis (T) tissue was used as a positive control. M: marker (161-0373; Bio-Rad Laboratories); d.: day after estrus; C: cyclic; P: pregnant; E: endometrial tissue (the reference sample applied in each blot); X: unrelated samples (randomly selected samples applied in marginal paths to avoid excessive bending of bands at the edge of gels). The red dashed lines show the fragment of each blot presented in Figure 2.

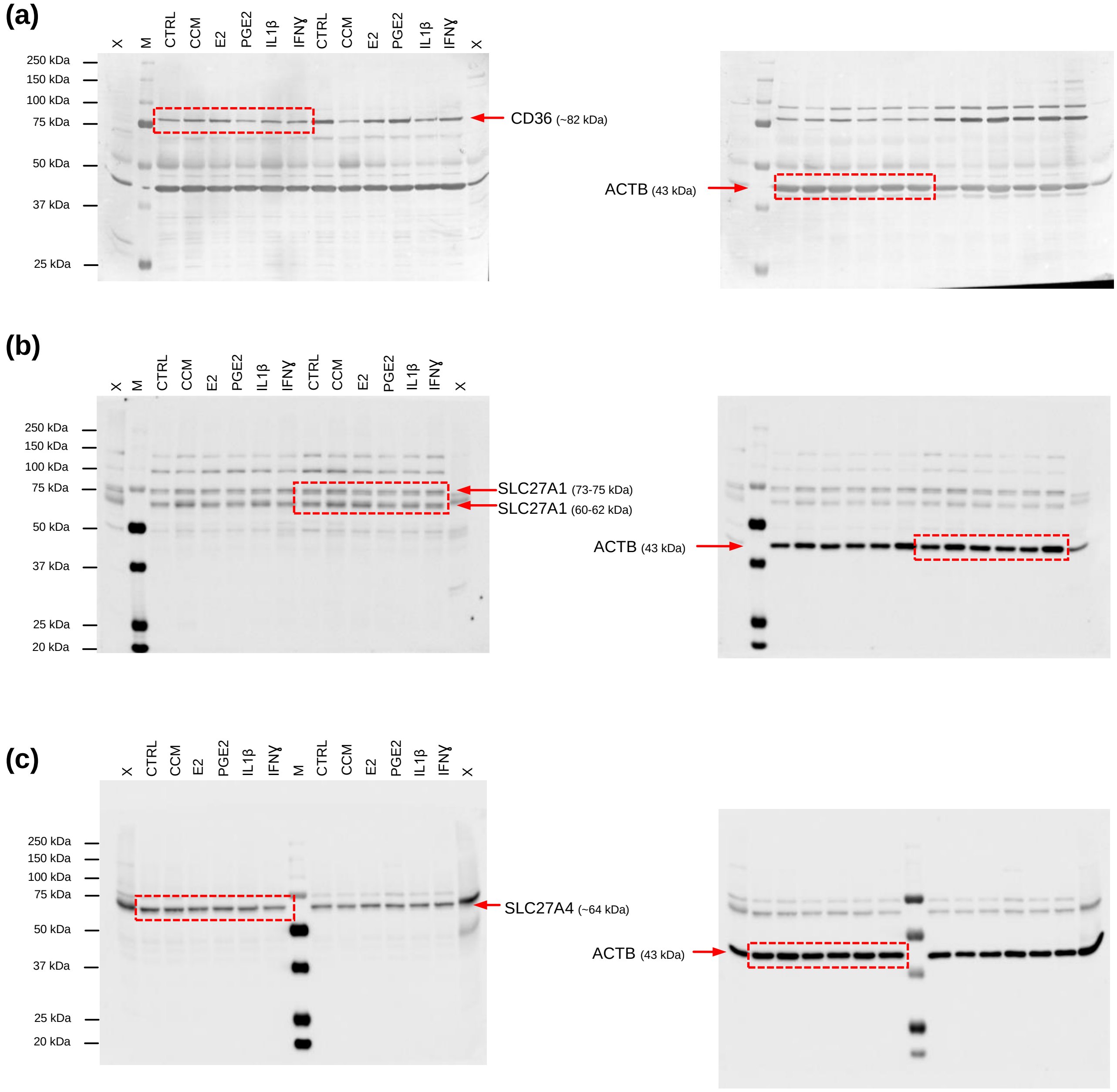

Supplementary Fig. S3. Western blot results presenting CD36 (**a**), SLC27A1 (**b**), and SLC27A4 (**c**) protein expression in endometrial slices exposed to conceptus-conditioned medium (CCM), estradiol-17β (E2), prostaglandin E2 (PGE2), interleukin 1β (IL1β), and interferon γ (IFNγ; left panel). β-actin (ACTB) protein was used as an internal control of protein loading. After being photographed, blots presented in the left panel were re-probed for ACTB and photographed again. CTRL: control; M: marker (161-0373; Bio-Rad Laboratories); X: unrelated samples (randomly selected samples applied in marginal paths to avoid excessive bending of bands at the edge of gels). The red dashed lines show the fragment of each blot presented in Figure 5.

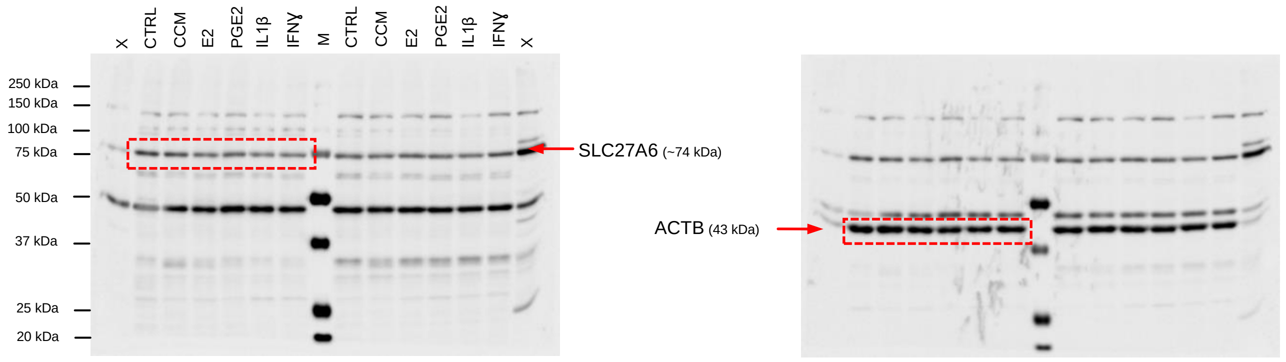

Supplementary Fig. S4. Western blot results presenting SLC27A6 protein expression in endometrial slices exposed to conceptus-conditioned medium (CCM), estradiol-17β (E2), prostaglandin E2 (PGE2), interleukin 1β (IL1β), and interferon γ (IFNγ; left panel). β-actin (ACTB) protein was used as internal control of protein loading (right panel). After being photographed, the blot presented in the left panel was re-probed for ACTB and photographed again. CTRL: control; M: marker (161-0373; Bio-Rad Laboratories); X: unrelated samples (randomly selected samples applied in marginal paths to avoid excessive bending of bands at the edge of gels). The red dashed lines show the fragment of each blot presented in Figure 5.

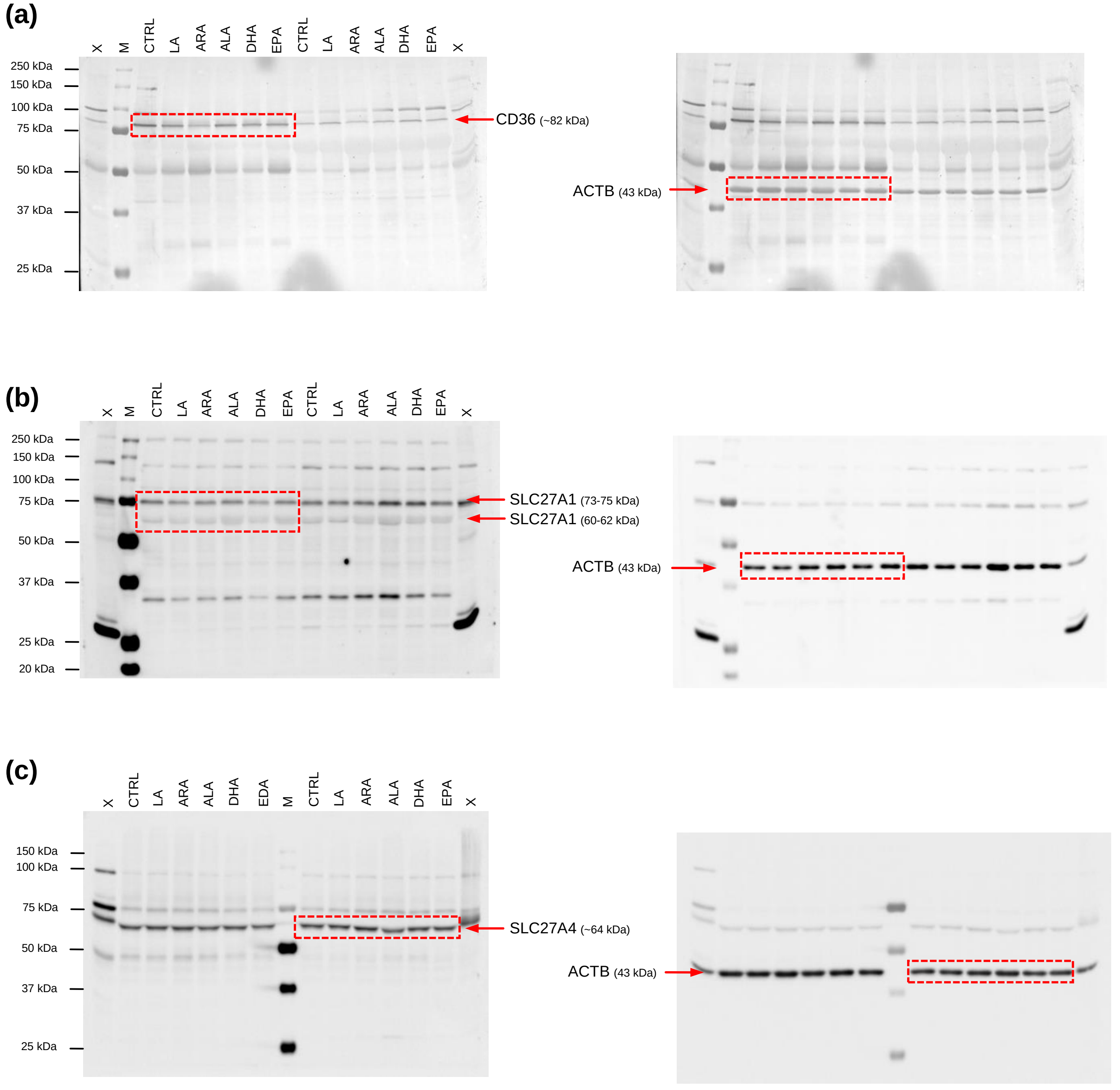

Supplementary Fig. S5. Western blot results presenting CD36 (**a**), SLC27A1 (**b**), and SLC27A4 (**c**) protein expression in endometrial slices exposed to linoleic acid (LA), arachidonic acid (ARA), α-linolenic acid (ALA), docosahexaenoic acid (DHA), or eicosapentaenoic acid (EPA; left panel). β-actin (ACTB) protein was used as an internal control of protein loading (right panel). After being photographed, blots presented in the left panel were re-probed for ACTB and photographed again. CTRL: control; M: marker (161-0373; Bio-Rad Laboratories); X: unrelated samples (randomly selected samples applied in marginal paths to avoid excessive bending of bands at the edge of gels). The red dashed lines show the fragment of each blot presented in Figure 6.

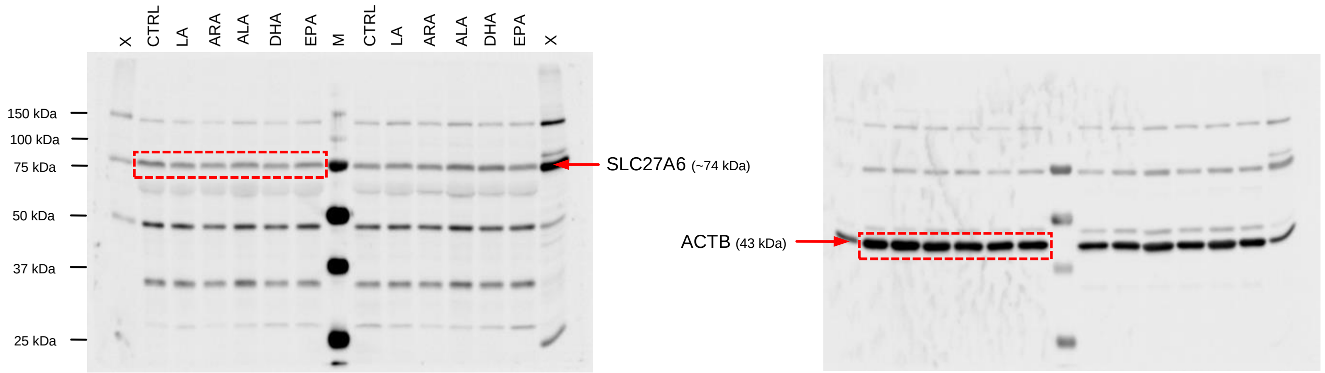

Supplementary Fig. S6. Western blot results presenting SLC27A6 protein expression in endometrial slices exposed to linoleic acid (LA), arachidonic acid (ARA), α-linolenic acid (ALA), docosahexaenoic acid (DHA), or eicosapentaenoic acid (EPA; left panel). β-actin (ACTB) protein was used as an internal control of protein loading (right panel). After being photographed, the blot presented in the left panel was re-probed for ACTB and photographed again. CTRL: control; M: marker (161-0373; Bio-Rad Laboratories); X: unrelated samples (randomly selected samples applied in marginal paths to avoid excessive bending of bands at the edge of gels). The red dashed lines show the fragment of each blot presented in Figure 6.

(a)

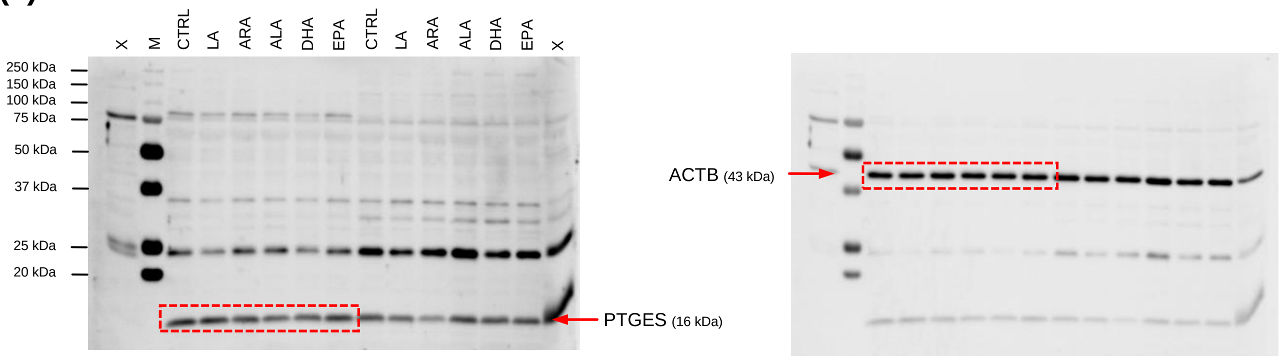

(b)

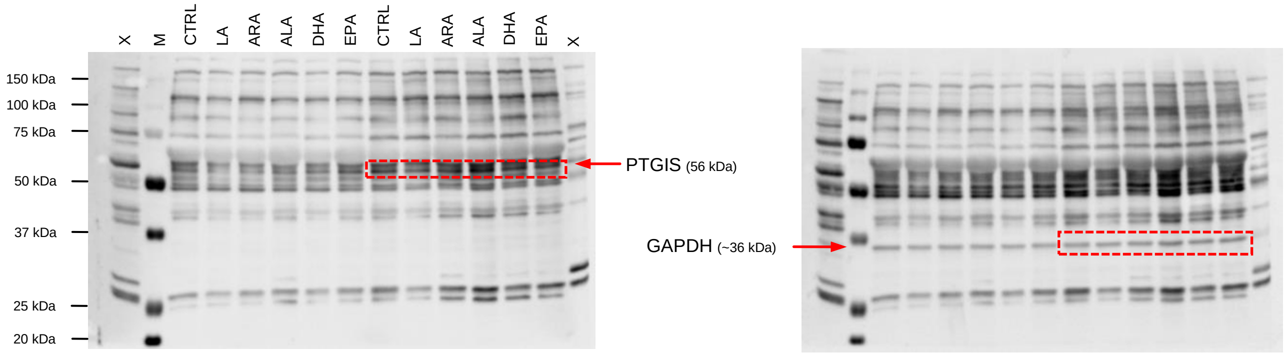

Supplementary Fig. S7. Western blot results presenting PTGES (**a**) and PTGIS (**b**) protein expression in endometrial slices exposed to linoleic acid (LA), arachidonic acid (ARA), α-linolenic acid (ALA), docosahexaenoic acid (DHA), or eicosapentaenoic acid (EPA; left panel). β-actin (ACTB) and glyceraldehyde-3-phosphate dehydrogenase (GAPDH) proteins were used as internal controls of protein loading (right panel). After being photographed, blots presented in the left panel were re-probed for ACTB or GAPDH and photographed again. CTRL: control; M: marker (161-0373; Bio-Rad Laboratories); X: unrelated samples (randomly selected samples applied in marginal paths to avoid excessive bending of bands at the edge of gels). The red dashed lines show the fragment of each blot presented in Figure 7.

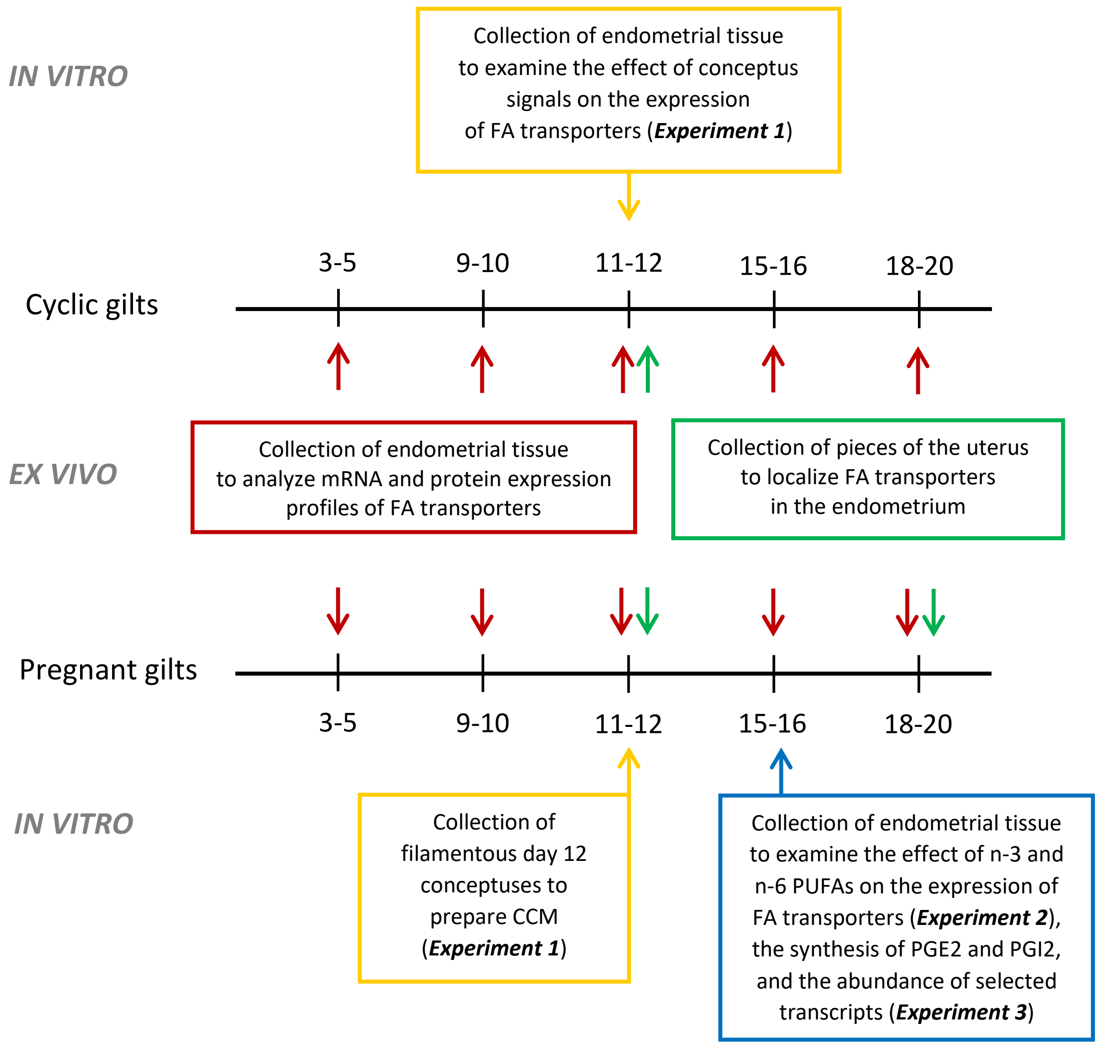

Supplementary Fig. S8. A schematic presentation of the study. Endometrial tissue or pieces of the uterus were collected from cyclic and pregnant gilts on days 3-5, 9-10, 11-12, 15-16, and 18-20 after ovulation and used for *ex vivo* analyzes and *in vitro* experiments. FA: fatty acid; PUFAs: polyunsaturated fatty acids; CCM: conceptus conditioned medium; PGE2: prostaglandin E2; PGI2: prostacyclin.
