# Supplementary material for: Expression Profiles of Fatty Acid Transporters and the Role of n-3 and n-6 Polyunsaturated Fatty Acids in the Porcine Endometrium
Source: Int J Mol Sci. 2024 Oct 16;25(20):11102. doi: 10.3390/ijms252011102 (PMC11507490; doi:10.3390/ijms252011102)
Supplement: Supplementary file 1 [file ijms-25-11102-s001.zip › Blitek_and_Szymanska_IJMS_Supplementary_Tables.pdf]

Supplementary Table S1. Full names of genes and the ID numbers of TaqMan probes applied to examine the relative mRNA expression using Real-time PCR**.**

Abbreviation	Gene name	ID of TaqMan probe
CD36	CD36 molecule; fatty acid translocase	Ss03388549_m1
SLC27A1	solute carrier family 27 member 1	Ss03388764_m1
SLC27A2	solute carrier family 27 member 2	Ss03373885_m1
SLC27A3	solute carrier family 27 member 3	Ss06872017_g1
SLC27A4	solute carrier family 27 member 4	Ss04329561_m1
SLC27A6	solute carrier family 27 member 6	Ss06904004_m1
PTGES	prostaglandin E synthase	Ss03392129_m1
PTGIS	prostaglandin I2 synthase	Ss03374149_m1
FABP3	fatty acid binding protein 3	Ss03386206_g1
FABP4	fatty acid binding protein 4	Ss03373313_m1
FABP5	fatty acid binding protein 5	Ss03392151_m1
PPARA	peroxisome proliferator activated receptor alpha	Ss03380164_u1
PPARD	peroxisome proliferator activated receptor delta	Ss03394198_g1
PPARG	peroxisome proliferator activated receptor gamma	Ss03394829_m1
ACOX1	acyl-CoA oxidase 1	Ss03386405_u1
CPT1A	carnitine palmitoyltransferase 1A	Ss03373366_m1
VEGFA	vascular endothelial growth factor A	Ss03393993_m1
FGF2	fibroblast growth factor 2	Ss03375809_u1
IL1B	interleukin 1 beta	Ss04321151_m1
IL6	interleukin 6	Ss07308316_g1
TNF	tumor necrosis factor	Ss03391318_g1
HPRT1	hypoxanthine phosphoribosyltransferase 1; reference gene	Ss03388274_m1
GAPDH	glyceraldehyde-3-phosphate dehydrogenase; reference gene	Ss03375435_u1
ACTG1	actin gamma 1; reference gene	Ss03376081_u1

Supplementary Table S2. Primary antibodies used in western blot (WB) analysis and immunohistochemical (IHC) or immunofluorescent (IF) procedures.

Protein	Name of antibody	Catalog no., manufacturer	The host species	Dilution used (analysis)
CD36	CD36/SR-B3 Rabbit pAb	A5792 / abclonal	rabbit	1:100 (WB)
	Scavenger Receptor B2/CD36 Polyclonal Antibody	100011 / Cayman Chemical	rabbit	1:500 (IF)
SLC27A1	SLC27A1 Rabbit pAb	A12847 / abclonal	rabbit	1:1000 (WB)<br>1:300 (IHC)
SLC27A4	FATP4/SLC27A4 Rabbit pAb	A16101 / abclonal	rabbit	1:100 (IHC)
	Anti-SLC27A4/FATP4 antibody [EPR17319] – C terminal	ab199719 / abcam	rabbit	1:1000 (WB)
SLC27A6	Anti-SLC27A6	SAB2102195-100UL / Sigma-Aldrich	rabbit	1:50 (IHC)
	SLC27A6 Rabbit pAb	A24512 / abclonal	rabbit	1:500 (WB)
PTGES	Prostaglandin E Synthase-1 (microsomal) Polyclonal Antibody	160140 / Cayman Chemical	rabbit	1:200 (WB)
PTGIS	Prostaglandin I Synthase Polyclonal Antibody	160640 / Cayman Chemical	rabbit	1:200 (WB)
ACTB	Anti-beta Actin antibody	ab8227 / abcam	rabbit	1:2000 (WB)
GAPDH	GAPDH Rabbit pAb	AC001 / abclonal	rabbit	1:1000 (WB)
